# *Mycobacterium abscessus* Mutants with a Compromised Functional Link between the Type VII ESX-3 System and an Iron Uptake Mechanism Reliant on an Unusual Mycobactin Siderophore

**DOI:** 10.3390/pathogens11090953

**Published:** 2022-08-23

**Authors:** Glennon V. Bythrow, Manal F. Farhat, Keith Levendosky, Poornima Mohandas, Gabrielle A. Germain, Barney Yoo, Luis E. N. Quadri

**Affiliations:** 1 Department of Biology, Brooklyn College, City University of New York, 2900 Bedford Avenue, Brooklyn, NY 11210, USA; 2 Biology Program, Graduate Center, City University of New York, 365 Fifth Avenue, New York, NY 10016, USA; 3 Department of Chemistry, Hunter College, City University of New York, 695 Park Avenue, New York, NY 10065, USA; 4 Biochemistry Program, Graduate Center, City University of New York, 365 Fifth Avenue, New York, NY 10016, USA

**Keywords:** *Mycobacterium abscessus*, nontuberculous mycobacteria, type VII secretion system, ESX-3, siderophore, mycobactin, iron uptake

## Abstract

The opportunistic pathogen *Mycobacterium abscessus* subsp. *abscessus* (*Mab*) has become an emerging public health threat due to the increasing number of *Mab*-associated chronic pulmonary disease cases. Treatment requires multiple drug courses and is often combined with surgical resection. Cure rates are only ~50% due to treatment failure and comorbidities. Deeper understanding of the biology of *Mab* is required to illuminate potential avenues for the development of better therapeutics against *Mab* infections. The ESX-3 type VII protein secretion system of *Mab* has an important role in host inflammatory and pathological responses during infection. In this work, we demonstrate a functional link between ESX-3 and an iron uptake system based on an unusual mycobactin-type siderophore (designated MBT Ab) and exploit this link to implement a large screen for transposon mutants with an impaired ESX-3. Most mutants we identified carry insertions in genes encoding predicted ESX-3 secretion machinery components or potential ESX-3 substrates. The mutants overproduce MBT Ab, a trait consistent with an iron uptake defect. Our characterization of MBT Ab revealed structural features reminiscent of nocardial mycobactin-like compounds with cytotoxicity. This finding raises the possibility that MBT Ab may play roles in pathogenesis unlinked to iron homeostasis. The mutants generated herein will facilitate research to better understand the role of ESX-3 and its interplay with the siderophore system.

## 1. Introduction

*Mycobacterium abscessus* subsp. *abscessus* (*Mab*), the best-known member of the *M. abscessus* complex, is a ubiquitous opportunistic nontuberculous mycobacterial pathogen responsible for community-acquired and healthcare-associated infections [1,2,3,4,5,6,7,8]. *Mab* has become an emerging public health menace due to the rising number of *Mab*-associated tuberculosis-like chronic pulmonary disease cases [1,3,4,5,6,8]. *Mab* is considered the most pathogenic of the rapidly growing *Mycobacterium* species and accounts for 65–80% of the chronic pulmonary disease cases associated with this group [1,3,4,5,6]. Patients with *Mab*-associated chronic pulmonary disease often have underlying risk factors such as cystic fibrosis, chronic obstructive pulmonary disease, previous mycobacterial lung infections, lipoid pneumonia, lung transplantation, or cancer [9,10,11,12]. The pulmonary disease caused by *Mab* is a daunting therapeutic challenge. Despite aggressive drug regimens, patients with *Mab*-associated chronic pulmonary disease are seldom cured (30–50% cure rate) due to drug treatment failure [13,14]. A deeper knowledge of the biology of *Mab* is required to illuminate potential avenues for the development of better therapeutics against *Mab* infections.

Recently, considerable attention has been given to conserved mycobacterial type VII protein secretion systems as potential targets for the development of tuberculosis drugs with novel mechanisms of action [15,16,17,18]. These systems are encoded by six paralogous chromosomal loci, known as *esx*-1, -2, -3, -4, -5, and -4-bis/-4_EV_. The number of *esx* loci present in each species varies. For example, there are five loci in *Mycobacterium tuberculosis* (*Mtb*) (*esx*-1 through -5), four loci in *Mycobacterium avium* (*esx*-2 through -5), three loci in *Mycobacterium leprae* (*esx*-1, -3, and -5), and two loci in *Mab* (*esx*-3 and -4) [17,19,20,21]. The heterogeneous distribution of ESX paralogues in mycobacteria raises the possibility of species-specific functional redundancies, specializations, and crosstalk between ESX systems. This scenario underscores the need for species-focused studies of these complex mycobacterial protein secretion systems.

Notably, *esx*-3 appears to be the only *esx* locus ubiquitous in mycobacteria of clinical significance [17]. A study aimed at discovering novel tuberculosis drug targets validated the EccB3 component of *Mtb* ESX-3 as a target candidate not exploited by existing tuberculosis drugs [16]. Experimental data accumulated by various studies indicate that ESX-3 is essential for *Mtb* growth under standard culturing conditions in iron-rich Middlebrook (MB) media and involved in mycobactin (MBT)/carboxymycobactin (cMBT) siderophore-mediated iron uptake, heme utilization, zinc homeostasis, immune response modulation, and virulence via both iron acquisition-dependent and iron acquisition-independent modalities [22,23,24,25,26,27,28,29,30,31,32,33,34]. The essentiality of *Mtb* ESX-3 can be bypassed by supplementing the MB media with a suitable alternative source of iron (i.e., an excess of hemin or MBT J–Fe^3+^ siderophore complex) [33]. The peculiar conditional essentiality of *Mtb* ESX-3 places the system in the group of conditionally essential target candidates for antimicrobial drug development [35].

The ESX-3 deficient *Mtb* mutant cultured in the growth-permissive medium experiences a deficiency of iron attributed to a defect in the utilization of MBT/cMBT-bound iron. Under this culture condition, the mutant displays a distinct orange pigmentation (OP) phenotype due to an excessive accumulation of MBT/cMBT–Fe^3+^ complexes [33]. The *Mtb* proteins Pe5 and Ppe4 encoded in the *esx*-3 locus are secreted by ESX-3 and thought to be implicated in the utilization of MBT/cMBT-bound iron and virulence in an iron acquisition-dependent manner [33]. On the other hand, the *Mtb* ESX-3 substrates Pe15 and Ppe20, which are encoded outside the *esx*-3 locus, are believed to be involved in virulence in an iron-uptake independent manner [33]. Overall, the emerging picture links the essentiality of *Mtb* ESX-3 to its critical function in the utilization of MBT/cMBT-bound iron and indicates that the secretion system plays multiple roles in *Mtb* biology and host–pathogen interaction that are not well understood.

Recent studies describing a *Mab* strain (Δ*esx*-3) with a 14-kb chromosomal deletion including the *esx*-3 locus (*MAB_2224c* through *MAB_2234c*) and a genome-wide analysis of gene essentiality revealed that *Mab* ESX-3 is not essential for growth under routine laboratory culturing conditions (standard, iron-rich MB media) [36,37]. The dispensability of *Mab* ESX-3 under standard culturing conditions contrasts with the essential nature documented for its counterpart in *Mtb*. However, paralleling the observations made in *Mtb*, *Mab* ESX-3 plays an important role in pathogenesis [36]. The *Mab* Δ*esx*-3 mutant has impaired survival in human macrophages and causes less pathology in mice than the wild-type (WT) strain [36]. However, *Mab* ESX-3’s involvement in the utilization of siderophore-bound iron has not yet been determined. Moreover, the production of MBT/cMBT-type siderophores by *Mab* has not been validated thus far. Isolation of presumptive MBT-type siderophores from *Mycobacterium chelonae* subsp. *abscessus* strains was reported almost thirty years ago [38,39]. However, the strains used in these early studies have unverifiable correspondence to contemporary *Mab* strains, including the type strain ATCC 19977^T^ [40] used in most laboratory studies and herein. We have recently highlighted the presence of orthologues of the genes encoding the nonribosomal peptide synthetases, polyketide synthases, and other proteins involved in the synthesis of MBT/cMBT siderophores of *Mtb* and *Mycobacterium smegmatis* (*Msm*) [41,42] in *Mab* ATCC 19977^T^ and other species [43]. The presence of these orthologues in *Mab* suggests that the bacterium has the capacity to produce an MBT-type siderophore. Overall, it is clear that additional studies on the ESX-3 and siderophore systems of *Mab* are warranted.

In this work, we demonstrate a functional link between the ESX-3 and siderophore systems of *Mab* and exploit this link to identify mutants with an impaired ESX-3. We also show that *Mab* produces an unusual MBT-type variant with features reminiscent of nocardial MBT-like compounds with cytotoxicity or antiproliferative activity. Our findings provide further insight into the ESX-3 and siderophore systems of *Mab* and underline the differences between the iron-acquisition capabilities of the opportunistic pathogen and *Mtb*. The collection of novel *Mab* mutants generated in this study will facilitate future research to better understand the functional dimensions of ESX-3 and its interplay with the siderophore system in *Mab*.

## 2. Results

### 2.1. Principle and Validation of the Screen for ESX-3-Impaired Mutants and Siderophore Production Probe

**Figure 1:**
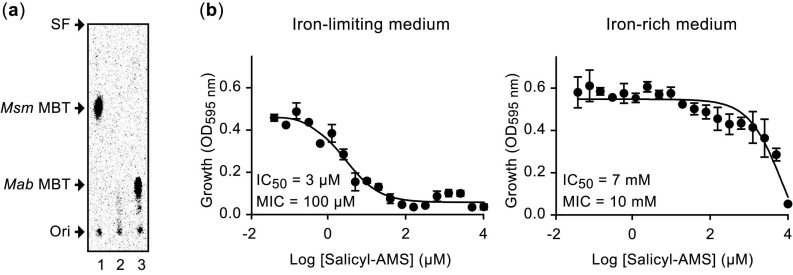
Radio-thin layer chromatography probe for presumptive mycobactin (MBT)−type siderophore in *M. abscessus* and antimicrobial activity of salicyl-AMS. (**a**) Thin layer chromatography (TLC) analysis. Lanes 1, 2, and 3 are samples from *M. smegmatis* (*Msm*), *Msm* Δ*mbtA* (MBT deficient mutant), and *M. abscessus* (*Mab*), respectively. The image shows the entire TLC plate. Solvent system: petroleum ether–n–butanol–ethyl acetate (2:3:3). Ori, origin; SF, solvent front. (**b**) Dose–response curves showing susceptibility of *Mab* to salicyl-AMS in GAST (iron-limiting) and GAST+Fe (iron-rich) media. The data represent means and standard deviations of triplicate cultures.

As noted above, *Mtb* ESX-3 mutants cultured under growth-permissive conditions that bypass the essentiality of ESX-3 display a distinct orange pigmentation (i.e., OP) phenotype caused by excessive accumulation of siderophore–Fe^3+^ complexes. This observation led us to speculate whether ESX-3 mutants of *Mab*, in which the secretion system is not essential [36], would display OP on s7H11 plates, and whether, if so, such a phenotype could be used to screen libraries of Tn mutants for strains with a dysfunctional ESX-3 or other defects leading to OP. Before exploring these ideas, we probed the capacity of *Mab* to produce a presumptive MBT-type siderophore. We previously reported an orthology analysis of MBT/cMBT biosynthesis genes that highlighted the potential of *Mab* to produce an MBT-like siderophore [43]. These genes include *mbtA*, which encodes the predicted salicylic acid-specific adenylation enzyme (MbtA) that catalyzes the first committed step in MBT/cMBT backbone biosynthesis [42,43]. We have also shown that exogenous [^14^C]salicylic acid can be utilized for biosynthesis of radiolabeled MBTs/cMBTs in *Mtb* and *Msm* cultures [42,43,44]. We used this labeling approach herein to probe the ability of *Mab* cultures to produce presumptive salicylic acid-derived siderophores by radio-TLC analysis. The results of this experiment revealed the production of a salicylic acid-derived compound (a presumptive MBT-type siderophore) with a retention factor (R_f_) different from the R_f_ of the MBT from *Msm* (Figure 1a). When the results of the TLC analysis are interpreted in the context of the published *Mtb* and *Msm* work, they suggest that *Mab* may indeed produce an MBT variant, an inference confirmed by the MS analysis described below.

We have previously shown that the rationally designed *Mtb* MbtA inhibitor salicyl-AMS suppresses MBT/cMBT biosynthesis and has potent antimicrobial activity against *Mtb* and *Msm* (strain producing only MBT/cMBT siderophores) conditional to culturing in iron-limiting medium, where the siderophores are needed for growth [44,45]. We assessed the antimicrobial activity of the inhibitor against *Mab*. The analysis revealed robust antimicrobial activity conditional to culturing in iron-limiting medium (iron-rich IC_50_ to iron-limiting IC_50_ ratio ~2000; iron-rich MIC to iron-limiting MIC ratio ~100) (Figure 1b). The activity of salicyl-AMS against *Mab* paralleled the effect of the inhibitor against *Mtb* and *Msm* [44,45]. The findings of the antimicrobial testing against *Mab* suggest that a siderophore is likely to be particularly critical for the bacterium’s growth in iron-limiting medium.

**Figure 2:**
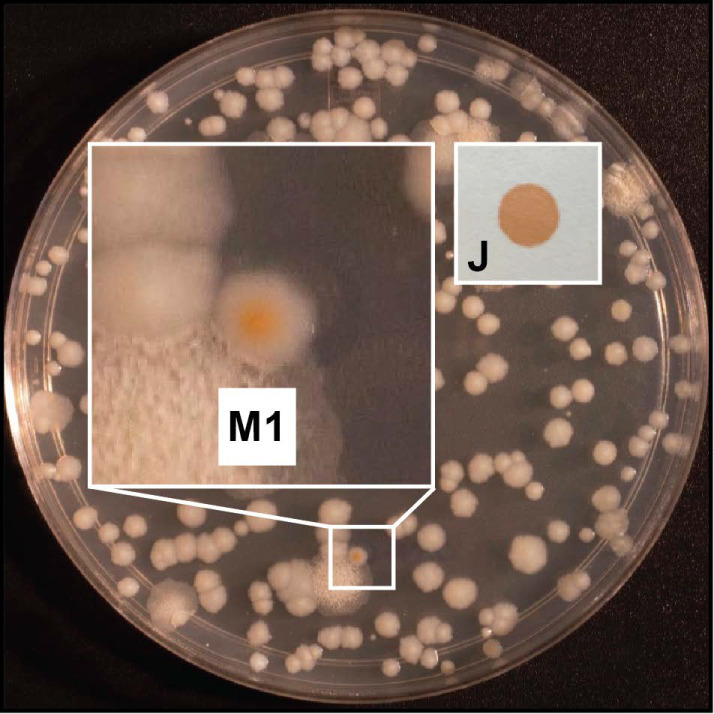
Plate of the pilot screen with the colony of isolate M1. The inset (labeled M1) shows the enlarged image of the plate section containing the colony of M1 (5.4× magnification). An image (labeled J) of an aliquot of ferric MBT J solution spotted on filter paper is shown to the right of the inset.

**Figure 3:**
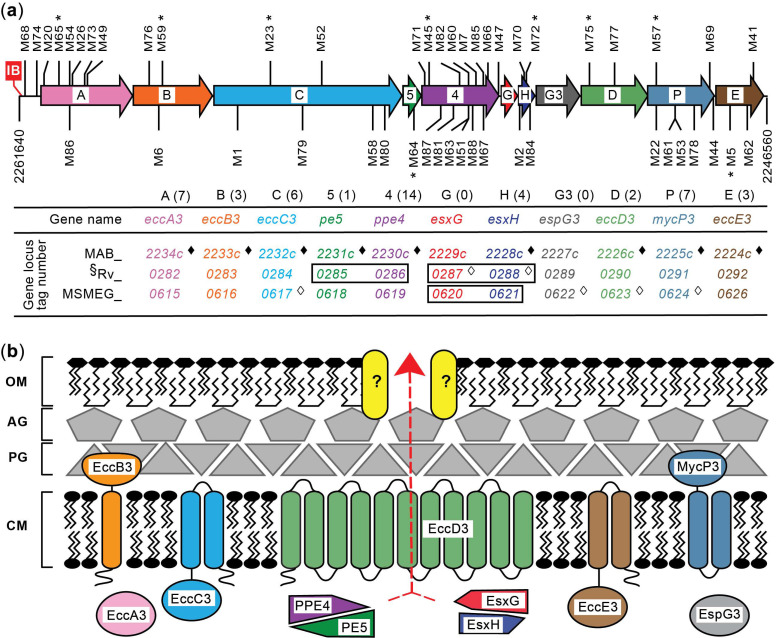
The ESX-3 system and its genetic dissection. (**a**) Schematic representation of the *esx*-3 locus of *M. abscessus* (*Mab*) and its transposon (Tn) mutants with OP. Genes are depicted as arrows labeled according to the single letter or number key shown below the gene cluster diagram. The number of insertion mutants isolated for each gene is given in parentheses. The 5′ and 3′ chromosomal coordinates of the genomic segment depicted are indicated. Tn insertions with the zeo^R^ gene in the same and opposite orientations relative to the *Mab* genes are marked above and below the locus, respectively. The Tn mutants marked with an asterisk (*) are those displayed in Figure 4. The location of a predicted iron box (IB) sequence (shown in Figure 5) for IdeR binding upstream *eccA3* is represented. Gene names noted for the predicted canonical ESX-3 substrates (*pe5*, *ppe4*, *esxG*, and *esxH*) and the conserved system components involved in substrate secretion (*eccA3*-*eccD3*, *espG3*, and *mycP*) are as per standard *esx* gene nomenclature [21]. Locus tags for the *Mab* genes (MAB_) and their orthologues in *M. tuberculosis* (*Mtb*, Rv_) and *M. smegmatis* (*Msm*, MSMEG_) are shown. *Mab esx-3* locus genes hit by the Tn in our collection of mutants are marked with a filled diamond symbol (♦). *Mtb* and *Msm* genes for which individual knockouts have been reported are marked with an open diamond symbol (◊) [26,33]. The gene pairs boxed represent reported double-gene deletions [26,33]. ^§^ *Mtb* genes listed are essential for growth on iron-rich 7H10 agar (except for *esxG* and *esxH*, for which mutation leads to a growth defect) by analysis of Himar1 Tn libraries [47]. (**b**) Assemblage model of ESX-3 components and substrates encoded in the archetypal *esx-3* locus. Two independent cryo-electron microscopy investigations of the *Msm* ESX-3 secretion complex have determined similar structures, composed of two dimerized core complexes each composed of one EccE, EccB, EccC, and two EccD components, in an organization simplified in the model depicted here. This ESX-3 dimer further trimerizes into a final hexamer structure similar to that seen for ESX-1 and ESX-5 complexes, forming the channel across which ESX-3 substrate components can be translocated. The fifth conserved ESX-3 membrane component, MycP, is not tightly associated with this described core secretion complex [48,49]. The yellow rounded rectangles (labeled with question marks) in the mycobacterial outer membrane layer (OM) signify the potential existence of protein(s) involved in facilitating the passage of ESX-3 substrates through the mycomembrane (adapted from [21,48,49]). AG, arabinogalactan layer; PG, peptidoglycan layer; CM, cytoplasmic membrane.

Having validated the production of a salicylic acid-derived compound (a presumptive MBT-type siderophore) in *Mab*, we proceeded to explore the screen concept. To this end, we generated a small pilot library of Tn mutants (~2500) and screened the library for colonies with OP on s7H11 plates. The pilot screen rendered one colony with OP (isolate M1). The orange coloration of M1 was clearly distinguishable from the off-white color of the rest of the colonies on the plates and resembled the color of an MBT J–Fe^3+^ standard [46] (Figure 2). Tn insertion site analysis indicated that the mutant had a single insertion (Figure S1), and it mapped to *eccC3* (*MAB_2232c*), the gene encoding the predicted core component EccC3 of the ESX-3 secretion machinery (Figure 3). By analogy with the *Mtb* ESX-3 mutant, we hypothesized that the OP of M1 (hereafter referred to as M1^eccC3^) is caused by accumulation of a siderophore–Fe^3+^ complex secondary to an ESX-3 malfunction.

Unexpectedly, during our initial experiments, we noticed a sporadic weakening or loss of the OP mutant phenotype on s7H11 plates. We eventually linked this puzzling phenomenon to changes in the lots of the commercial bovine serum albumin (BSA) used to prepare the standard ADN supplement added to 7H11. We found that BSA was needed in the agar to observe the OP phenotype (Figure S2a), and that some BSA lots supported the development of OP poorly or not at all. We also found that increasing the concentration of the BSA from such lots in the medium supplement from the standard 0.5% to 1.25–2.5% afforded reproducible OP development (Figure S2b). In view of these early findings, we subsequently prepared the ADN supplement with BSA at higher concentrations (typically 1.5%) depending on each BSA lot’s pre-assessed performance in supporting OP development by previously collected mutant strains. In all, the findings of our pilot experiments provided the first indication of a functional link between the ESX-3 and siderophore systems of *Mab*, validated the screening approach, and set the methodological stage for a large-scale screen.

### 2.2. Large-Scale Screen and Genetic Characterization of Isolates with OP Phenotype

The results described above encouraged us to scale up the screen. We screened a total of ~196,000 mutants, a library reaching an average of one Tn insertion per 26 bp of genomic DNA. This frequency corresponds to a theoretical probability of the Tn missing the reference ~1000-bp average size gene of *Mab* by chance of ~10^−16^ (Figure S3). Moreover, the library could be considered to be near-saturation (i.e., having mutants of nearly all nonessential genes represented) based on the observation that reaching near-saturation in bacterial Tn insertion libraries requires 6000–7000 mutants per megabase of genome [50,51,52]. This threshold was surpassed by ~6-fold in our library.

The screen led to the identification of 54 isolates with OP phenotype (including M1^eccC3^). The results of the Southern blot analysis confirmed a single insertion in 52 of the isolates (Figure S1). The remaining two isolates (M7 and M63) showed two hybridization bands that could have originated from two insertions or incomplete DNA digestion (not shown). However, both M7 and M63 had at least one insertion in an *esx-3* locus gene. The results of the insertion site determinations for all isolates are summarized in Figure 3a and Table 1. Of the 52 isolates with a single insertion, 47 carried an insertion in one of the eleven genes in the *esx-3* locus, and two had an insertion in a putative promoter region at the 5′-end of the locus (Figure 3). The remaining five isolates had insertions outside either the *esx-3* locus or its predicted promoter region (Table 1). Four of these insertions mapped to four different genes, i.e., *MAB_1912c*, *MAB_2276c*, *MAB_4275c*, and *MAB_4783*. The fifth insertion mapped to the putative promoter region of *MAB_4537c*. These five genes encode proteins of unknown function and different degrees of conservation across *Mycobacterium* species (Table S4). To our knowledge, there are no reports of functional links between any of these genes and ESX or siderophore systems, or iron or zinc homeostasis. Notably, however, *MAB_4783* encodes a paralogue of the predicted ESX-3 substrate Ppe4 (MAB_2230c; 37% amino acid identity) with close orthologues only present in the *M. abscessus* complex, *M. chelonae*, and four species closely related to the *M. chelonae*–*M. abscessus* group (Table S4).

### 2.3. Colony and Macrocolony Phenotypes of Mutant Isolates

**Figure 4:**
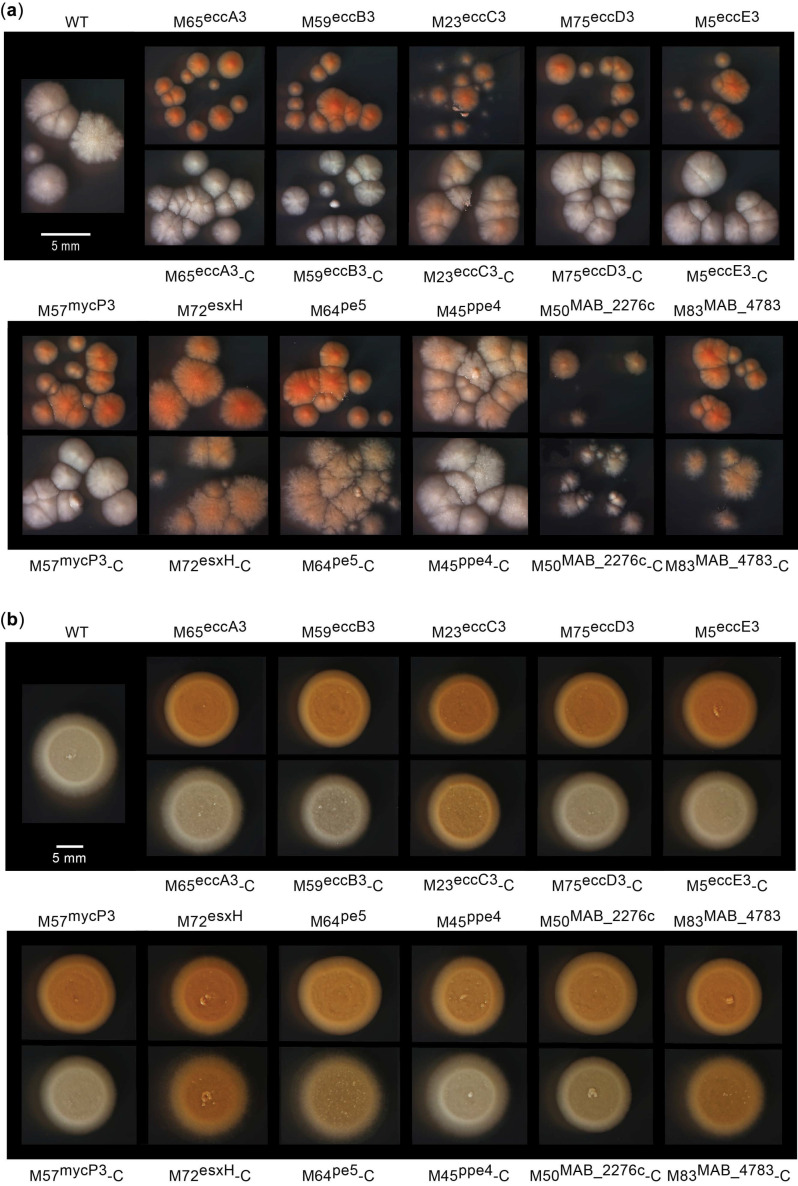
Phenotype of representative single colonies (**a**) and macrocolonies (**b**) of *M. abscessus* strains. Top and bottom rows in (**a**,**b**) depict the Tn mutants and their corresponding genetic complementation control strains, respectively. The wild-type strain (WT) and the mutants in the top rows carried pML1335 (empty), the vector used in the genetic complementation experiments, so that all strains could be grown in the same antibiotic-containing medium. Images of single colonies (7 days old) and spot inoculation-derived macrocolonies (5 days old; 1.5 µL inoculum, culture OD_600_ = 1.0) were digitally captured using an Olympus SZX7 stereo microscope (Olympus Corp., Center Valley, PA, USA) and a T2i DSLR camera (Canon Inc., Melville, NY, USA), respectively. Scale bars are shown.

Figure 4 shows the OP phenotype of single colonies and macrocolonies (arising from spot-inoculation of liquid cultures onto agar [54,55]) of nine representative *esx-3* locus mutants (M65^eccA3^, M59^eccB3^, M23^eccC3^, M75^eccD3^, M5^eccE3^, M57^mycP3^, M72^esxH^, M45^ppe4^, and M64^pe5^; highlighted in Figure 3), two mutants with an insertion outside the *esx-3* locus (M83^MAB_4783^ and M50^MAB_2276c^), and the respective genetic complementation control strain for each of the eleven mutants. Robust complementation was achieved for M65^eccA3^, M59^eccB3^, M75^eccD3^, M5^eccE3^, M57^mycP3^, and M45^ppe4^, as demonstrated by a drastic reduction in pigmentation intensity in single colonies (Figure 4a) and macrocolonies (Figure 4b) of their respective complementation control strains. Weak partial complementation was observed for the remaining five mutants (i.e., M23^eccC3^, M72^esxH^, M64^pe5^, M50^MAB_2276c^, and M83^MAB_4783^). The complementation controls of these mutants showed only a slight reduction in pigmentation intensity, which was more evident in single colonies (Figure 4a) than in macrocolonies (Figure 4b). Interestingly, the *esxH* mutant could be fully complemented by an *esxG*-*esxH* fragment (Figure S4). The remaining three mutants with insertions outside the *esx-3* locus (M56^MAB_1912c^, P5^MAB_4275c^, and M55^MAB_4537c^) did not show signs of complementation (not shown) and were not further investigated.

### 2.4. Sequence Bioinformatics Suggests the esx-3 Locus of M. abscessus Is Regulated in Response to Iron by IdeR

**Figure 5:**
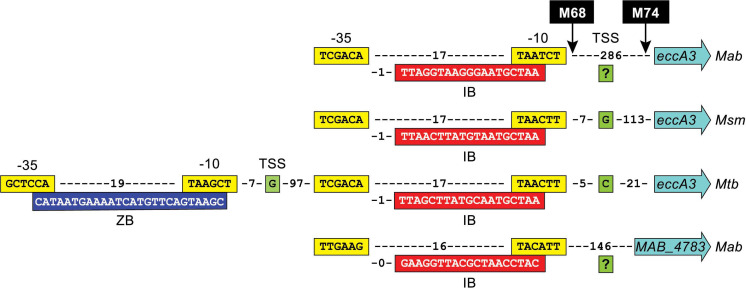
Diagram of the proposed promoter region of *M. abscessus* (*Mab*) *eccA3*, the orthologous promoter regions in *M. smegmatis* (*Msm*) and *M. tuberculosis* (*Mtb*), and the proposed promoter region of *MAB_4783*. Predicted -35 and -10 sequence elements (yellow boxes), binding sites for IdeR (IB, red boxes) and Zur (ZB, blue box), and transcription start sites (TSS, green boxes) are depicted. Genes are shown as arrows (light blue), with arrowheads indicating gene orientation. Numbers displayed between elements indicate the number of nucleotides between them. The information presented for the *Msm* and *Mtb eccA3* promoter regions is a compilation from reported studies [22,27,56,57,58]. The -35 and -10 elements and iron boxes in the promoters of *Mab eccA3* and *MAB_4783* were predicted as described in the Materials and Methods section. The iron boxes of *Mab eccA3* and *MAB_4783* correspond to putative iron boxes 24^C^ and 52 in Figure 6, respectively. The TSS of *Mab* genes (denoted by a boxed question mark) is unknown. The positions of the Tn insertions in mutant M68 (257 nucleotides away from the annotated start codon) and mutant M74 (6 nucleotides away from the annotated start codon) are depicted.

As noted above, M74^peccA3^ and M68^peccA3^ have an insertion in a putative promoter region upstream of *eccA3* (Figure 3, Table 1). A search for promoter elements in this region revealed potential -10 and -35 sequence motifs upstream of the Tn insertions (Figure 5). Notably, these sequence motifs differed by only two mismatches (-10 motif) from those in the promoters upstream of the *Mtb* and *Msm eccA3* orthologues. These findings are in line with the notion that disruption of the transcription of *esx-3* locus genes downstream of the Tn insertion in M74^peccA3^ and M68^peccA3^ causes the OP phenotype of the mutants. Future studies to probe the hypothesized basis of the phenotype observed in M74^peccA3^ and M68^peccA3^ are warranted.

**Figure 6:**
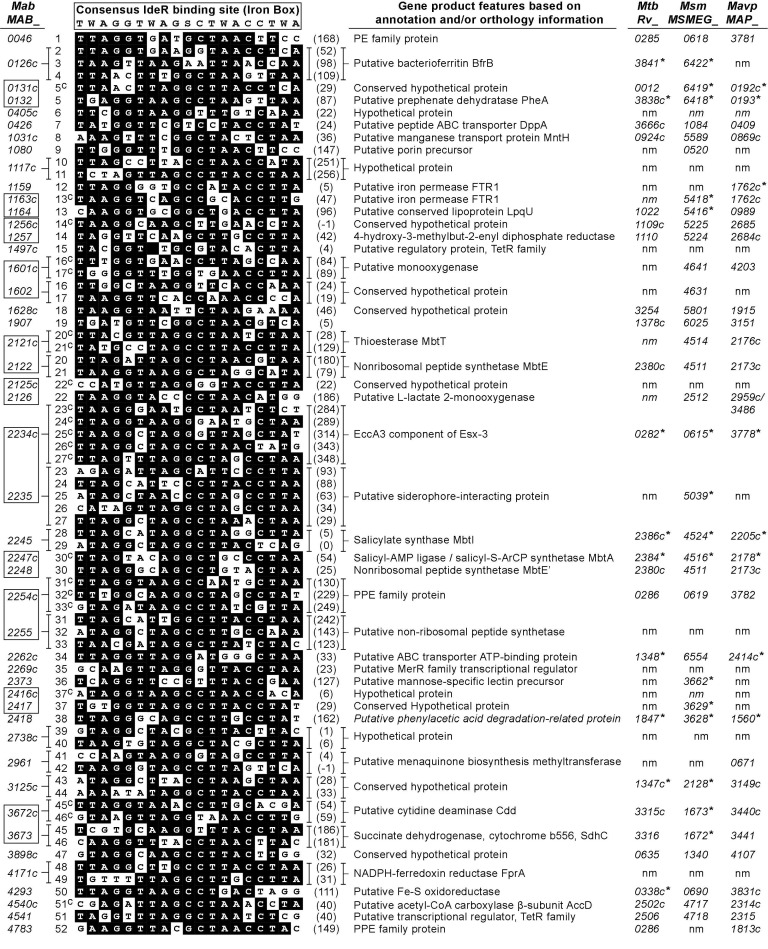
Putative IdeR binding sites in *M. abscessus.* All binding sites in the alignment are shown in the 5′ to 3′ direction. Bases on a black background match the iron box sequence consensus. The locus tags of annotated *M. abscessus* (*Mab*) genes located at up to 350 nucleotides downstream of the 3′ end of each iron box are shown to the left of the alignment. The number of bases between the 5′ end of each of these genes and the 3′ end of the iron box is shown in parentheses to the right of the alignment. Adjacent genes with divergent transcription orientation and potentially regulated by IdeR binding to the same site are boxed. The superscripted letter “c” adjacent to the iron box number displayed for one member in each of these boxed gene pairs indicates that the iron box is the reverse-complement of the iron box with the same number (e.g., 20^c^ is the reverse-complement of 20). Locus tags of predicted orthologues of *Mab* genes in *M. tuberculosis* (*Mtb*), *M. smegmatis* (*Msm*), and *Mycobacterium avium* subsp. *paratuberculosis* (*Mavp*) are shown. The nm notation in the orthology column indicates no orthologue match found. Orthologues marked by an asterisk (*) are reported to have an iron box (predicted or experimentally validated) in their respective promoter regions [57,58,59,60,61].

The promoter of the *Mab eccA3* orthologue in *Msm* (*MSMEG_0615*) is controlled by the iron-dependent regulator IdeR [22,56,58]. In contrast, the promoter of the *eccA3* counterpart in *Mtb* (*Rv0282*) is controlled by both IdeR and the zinc-dependent regulator Zur [22,27,56,58]. We searched the putative promoter region of *Mab eccA*3 for potential binding sites for IdeR (iron box) and for Zur (Zur box). The searches revealed a potential iron box with a location analogous to that of the iron boxes present in the *esx-3* loci of *Msm* and *Mtb* (Figure 5). The analysis, however, did not reveal a putative Zur box. We extended the iron box search to the rest of the *Mab* genome to identify other potential IdeR-regulated genes. The search revealed several iron boxes in the chromosome, many of which have counterparts in orthologous loci of other mycobacteria (Figure 6). Notably, a putative iron box was found in the predicted promoter regions of *MAB_4783* (the *ppe4* paralogue disrupted in M83^MAB_4783^) and the MBT biosynthesis genes *mbtT* (*MAB_2121c*), *mbtE* (*MAB_2122*), *mbtA* (*MAB_2247c*), *mbtE′* (*MAB_2248*), and *mbtI* (*MAB_2245*) [42,43] (Figure 6). These results support the idea that transcription of the *Mab esx-3* locus genes, *MAB_4783*, and the MBT biosynthesis genes might be co-regulated in response to intracellular iron levels by *Mab* IdeR (MAB_3029; 86% identity with *Mtb* IdeR). Overall, these findings strengthen the hypothesized physiological interplay between the ESX-3 and MBT siderophore systems of *Mab* noted above and underscore a role of *Mab* ESX-3 in iron homeostasis. Our findings also highlight directions for future studies on the expression levels of *esx*-3 and MBT biosynthesis genes and their hypothesized iron/IdeR-dependent regulation.

### 2.5. The Orange Pigmentation Phenotype of M. abscessus Cultures Is Influenced by Iron Availability

**Figure 7:**
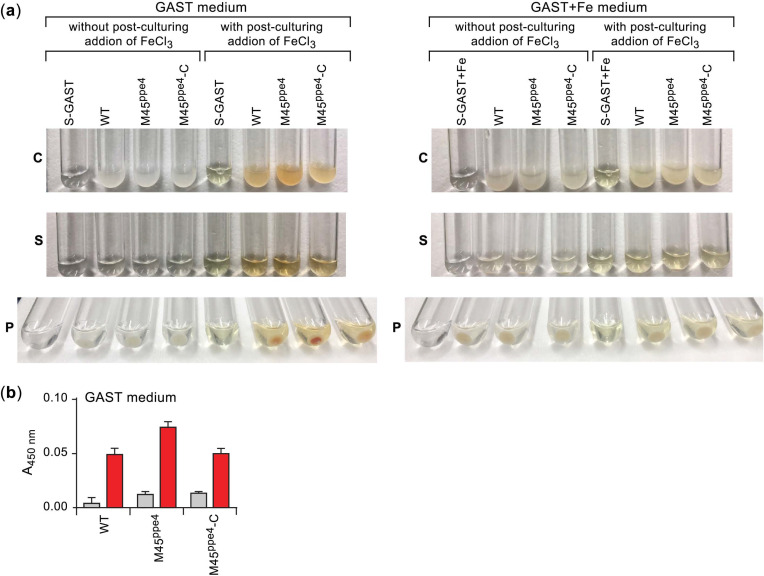
The orange pigmentation phenotype of *M. abscessus* cultures is influenced by iron availability. (**a**) Cultures (C), spent supernatants (S), and pellets (P) of strains grown to saturation in iron-limiting GAST broth (left panel) or iron-rich GAST+Fe broth (GAST supplemented with FeCl_3_ to 100 µM; right panel). After incubation for growth, cultures were treated by addition of FeCl_3_ (to 5 mM; right half of each panel) to allow maximal conversion of the colorless MBT into the orange MBT–Fe^3+^ complex, or left untreated (left half of each panel). S-GAST and S-GAST+Fe, sterile GAST and GAST+Fe broth controls, respectively. (**b**) Spectrophotometric quantification of MBT–Fe^3+^ in culture supernatants of strains grown to saturation in GAST broth, and then treated by addition of FeCl_3_ (to 5 mM; red bars) or left untreated (gray bars). A_450_, blank (sterile broth)-corrected absorbance at 450 nm. The data represent means ± SE from three cultures. The wild-type strain (WT) and M45^ppe4^ carried pML1335 (empty), the vector used in the genetic complementation experiments, so that they could be grown along with the complemented strain M45^ppe4^-C in the same antibiotic-containing medium.

Based on our analysis of putative promoter regions, we hypothesize that growth in iron-limiting conditions will cause de-repression of IdeR-regulated *esx-3* and MBT biosynthesis genes. In addition, we predict that the upregulation of MBT biosynthesis genes will lead to an increase in MBT accumulation in both WT and mutant cultures. To test this view, we probed for OP in cultures of selected *esx-3* mutants (M45^ppe4^, M57^mycP3^, M65^eccA3^, and M75^eccD3^) grown in GAST (iron-limiting, BSA free) and GAST+Fe (GAST supplemented with 100 µM FeCl_3_), two media where the mutants and the WT strain display comparable growth (see below). In line with our expectation, an OP analogous to that seen in the colonies and macrocolonies of the mutants was observed in the GAST cultures. While these cultures had no visible OP initially (presumably because restricted iron in the medium does not support substantial formation of the orange MBT–Fe^3+^ complex), post-culturing addition of excess FeCl_3_ (5 mM) to the cultures for maximal conversion of colorless MBT into MBT–Fe^3+^ led to an instantaneous development of OP in the WT and the four mutants tested; i.e., M45^ppe4^ and M57^mycP3^ (Figure 7a and Figure S5a), and M65^eccA3^ and M75^eccD3^ (not shown). The pigmentation was chiefly associated with the cells, as determined by visual inspection of both the cell pellets and spent culture supernatants (Figure 7a and Figure S5b). Nonetheless, the supernatants retained some pigmentation detectable by the naked eye and quantifiable by spectrophotometric analysis (Figure 7b and Figure S5b). The pigmentation of the *esx-3* mutants was markedly more intense than that observed in the WT strain (M45^ppe4^ and M57^mycP3^, Figure 7a and Figure S5a) or essentially indistinguishable from it (M65^eccA3^ and M75^eccD3^, not shown). In the former case, genetic complementation reduced the pigmentation intensity to WT levels. Growth of the WT and mutant strains in GAST+Fe, however, did not lead to visible pigmentation in any of the cultures, with or without post-culturing addition of excess FeCl_3_ (Figure 7a and Figure S5a). The findings with the GAST+Fe are in agreement with the hypothesized IdeR-dependent downregulation of MBT biosynthesis in both WT and mutant strains in the iron-rich medium.

### 2.6. Growth Characterization of esx-3 Locus Mutants, M83^MAB_4783^, and M50^MAB_2276c^

**Figure 8:**
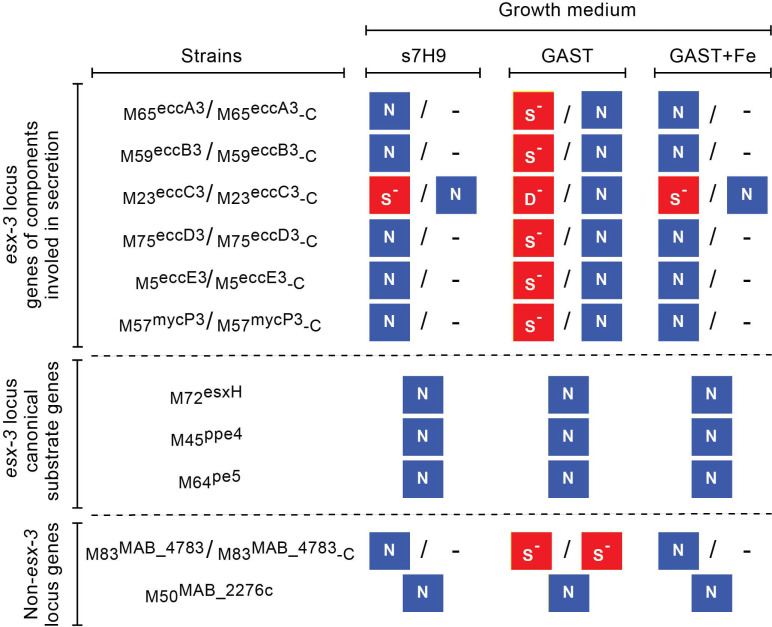
Impact of transposon insertions on bacterial growth in s7H9, GAST, and GAST+Fe media. No negative impact on growth (N) and negative impact on growth (S^−^, small; D^−^, drastic) are highlighted in blue and red backgrounds, respectively. The information presented summarizes the growth curve results shown in Figure S6. Strains are grouped in three sections according to the functional category and location of the gene disrupted by the transposon.

To gain further insight into the effect of the insertions in the mutants, we compared the growth of the WT strain and the representative mutants displayed in Figure 4 in standard s7H9 (iron rich), GAST (iron limiting), and GAST+Fe (iron rich) media. Figure 8 summarizes the results of growth curves shown in Figure S6. In s7H9, only the *eccC3* mutant had an appreciable growth delay compared to the WT strain. The defect was modest, and it was not present in the mutant’s complementation control strain (Figure S6a). In GAST, however, all *esx-3* locus mutants with insertions in genes of ESX-3 components predicted to be involved in secretion (*ecc* genes and *mycP3*) showed some degree of growth delay relative to the WT strain (Figure S6b). The delay was small for the *eccA3*, *eccB3*, *eccD3*, *eccE3*, and *mycP3* mutants, but drastic for the *eccC3* mutant. The growth of the complementation control strains of these six mutants was no different from that of the WT strain (Figure S6b). The growth defect of the *eccA3*, *eccB3*, *eccD3*, *eccE3*, and *mycP3* mutants was also absent in GAST+Fe (Figure S6c). In contrast, the growth defect of the *eccC3* mutant was not fully suppressed in GAST+Fe, where the mutant had an appreciable growth delay analogous to that seen in the iron-rich s7H9 medium. The defect was, however, eliminated by genetic complementation (Figure S6c).

Unlike the secretion machinery mutants noted above, the mutants with insertions in predicted ESX-3 substrate genes (i.e., *pe5*, *ppe4*, and *esxH* mutants) did not show a growth abnormality in GAST (Figure S6b). These mutants also had normal growth in GAST+Fe (Figure S6c). The medium-specific growth pattern of the *ppe4* mutant was not recapitulated in the *MAB_4783* mutant, which carried the insertion in a *ppe4* paralogue (Figure S6a–c). Both the *MAB_4783* mutant and its complementation control strain showed a slight growth delay in GAST (Figure S6b), a defect absent in GAST+Fe (Figure S6c). Lastly, the mutant with the insertion in *MAB_2276c* (encoding a putative regulatory protein, Table S4) had no growth delay in any media (Figure S6b,c). Overall, the growth characterization data indicate that insertions disrupting the ESX-3 secretion apparatus lead to a fitness cost conditional to (or in the case of *eccC3* exacerbated by) culturing in the iron-limiting medium. In contrast, insertions disrupting the predicted canonical ESX-3 substrate genes do not impact fitness in iron-limiting or iron-rich media.

### 2.7. Mass Spectrometry Analysis of M. abscessus Mycobactin

To gain insight into the structural features of the presumptive MBT-type siderophore of *Mab* (hereafter called MBT Ab), we undertook a siderophore-targeted, LC-MS/MS-based metabolomics approach. To this end, we explored the structure of the siderophore produced by the WT strain and the *mycP3* Tn mutant, which has a disruption of the gene encoding the predicted MycP (mycosin protease) component conserved in type VII secretion systems [18]. As noted above, the mutant displayed siderophore overproduction, and a robust OP phenotype on iron-rich s7H11 plates and iron-limiting broth readily complemented by a WT copy of the gene (Figure 4 and Figure S5). We grew both strains in iron-limiting broth to upregulate siderophore production, and processed cell pellets and spent culture supernatants for isolation of MBT-type siderophores. The samples were then subjected to high-resolution LC-MS/MS analysis using an iron isotope-assisted screen for iron-containing metabolites in the mass range expected for MBT/cMBT siderophores. LC-MS conditions were initially guided by protocols we previously developed for the siderophore of *Msm* [43], and then iteratively optimized for presumptive siderophores in *Mab* samples. Commercially available MBT J was used as a structural reference in the LC-MS/MS studies. Our explorative pilot experiments (not shown) revealed similar presumptive siderophore ions and MS/MS signatures in the WT and *mycP3*::Tn strains, and across samples from cell pellets and culture supernatants. The analysis showed that MBT J and the presumptive *Mab* siderophore exhibit similar MS/MS fragmentation patterns, while also sharing a few of the same daughter ions. The pilot experiments also demonstrated a higher total (summed) siderophore abundance in the mutant than in the WT strain, a finding consistent with the more intense OP of the *esx-3* mutant (Figure 7). In view of these results, we carried out our analysis to obtain a detailed structural characterization of the *Mab* siderophore with the more readily available siderophore obtained from the cells of the mutant.

**Figure 9:**
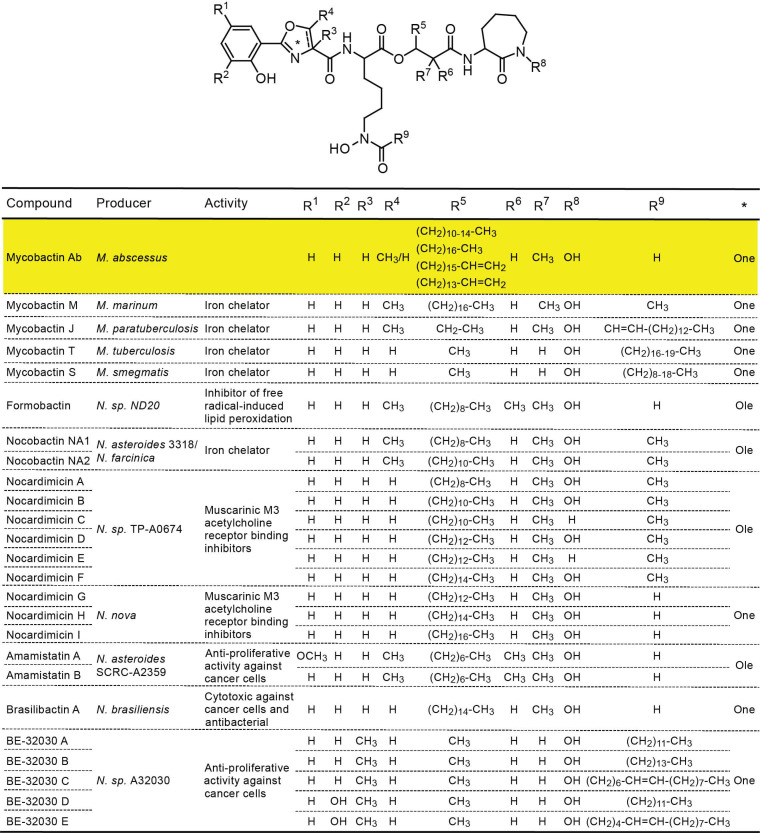
Mycobactin Ab (highlighted in yellow), other mycobactins, and related nocaridal metabolites. One, oxazoline; Ole, oxazole.

Based on the findings of LC-MS analysis (Figure S8), we propose the structure of the scaffold of MBT Ab shown in Figure 9. The figure also shows structural information for other mycobacterial siderophores and related compounds from *Nocardia* species reported in the literature [43,46,62,63]. Surprisingly, two unusual structural features differentiate MBT Ab from most previously characterized mycobacterial siderophore variants (e.g., *Mtb* and *Msm* MBTs, and MBT J) (Figure 9). First, MBT Ab does not have an acyl substituent on the Nε of the internal hydroxylysine residue. In other mycobacteria, the biosynthesis and addition of this substituent have been shown to require four genes (*mbtK*, *mbtL*, *mbtM*, *mbtN*) located in the so-called *mbt-2* gene cluster [64,65,66]. Our orthology analysis (not shown) and analysis by others [66] did not reveal an *mbt-2* locus orthologue in *Mab*. This finding is consistent with the lack of an acyl substituent on the internal hydroxylysine residue of MBT Ab. Second, MBT Ab has a long alkyl chain of variable length in the moiety predicted to be assembled by the MbtC (MAB_2120c)-MbtD (MAB_2119c) polyketide synthase system of the pathway [42,43]. MBT Ab shares structural features with a recently proposed structure of the MBT of *M. marinum* (MBT M [62]; Figure 9), which also lacks an acyl substituent on the internal hydroxylysine and has an alkyl chain with or without a terminal carboxylic acid functionality at the equivalent position of the variable alkyl chain of MBT Ab (Figure 9). Notably, the two salient structural features of MBT Ab noted above are common in several nocardial compounds with core scaffolds remarkably similar to those of MBTs [43] (Figure 9).

**Figure 10:**
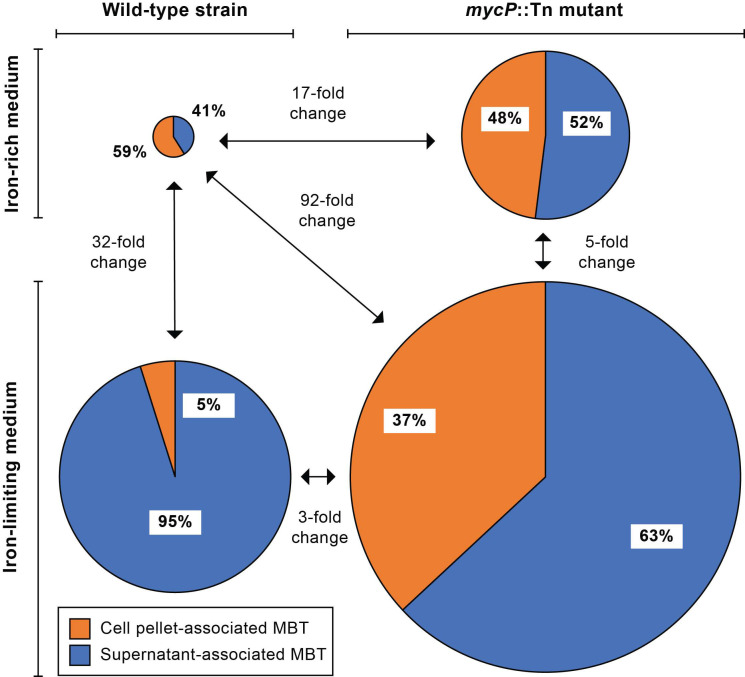
Impact of ESX-3 secretion machinery impairment and extracellular iron availability on MBT Ab abundance. Cultures of the wild-type strain and the *mycP3*::Tn mutant were grown in iron-limiting medium or iron-rich medium (iron-limiting medium supplemented with 100 µM FeCl_3_). Cell pellet-associated and culture supernatant-associated siderophores were extracted and analyzed by LC-MS. The pie charts show relative siderophore abundance obtained by ion peak integrations from extracted ion chromatograms. The areas of charts for the wild-type strain in iron-limiting medium (lower left pie; 32 cm^2^), the mutant strain in iron-limiting medium (lower right pie; 92 cm^2^), and the mutant strain in iron-rich medium (upper right pie; 17 cm^2^) are proportional to siderophore abundance relative to the total siderophore abundance of the wild-type in iron-rich medium (upper left pie; 1 cm^2^). The results shown represent aggregated data of abundance collected in duplicate for the same seven different MBT Ab structural variants for each sample.

Following the structure elucidation of MBT Ab, we used the LC-MS platform to investigate siderophore abundance in cultures of the WT and *mycP3*::Tn strains in iron-limiting and iron-rich growth media. To this end, we analyzed aggregated (summed) data of siderophore abundance determined by ion peak integrations from extracted ion chromatograms collected for seven different MBT Ab structural variants in samples of cell pellet-associated and supernatant-associated siderophore extracts (Table S5). The results of this analysis, which provides a more reliable picture than an analysis based on any single MBT Ab variant could afford, are shown in Figure 10. In the iron-rich condition, the ESX-3 mutant displayed a 17-fold increase in total MBT abundance relative to the WT strain reference. In contrast, the ratio of culture supernatant-associated MBT to cell pellet-associated MBT did not diverge drastically between the strains (1.6-fold change). In the iron-limiting condition, both strains had increased MBT abundance relative to their respective iron-rich condition reference. The increase was more drastic for the WT strain (32-fold) than for the mutant (5-fold), which had already higher MBT abundance in the iron-rich condition than the WT reference (17-fold). Notably, while there was a relatively modest 3-fold difference in total MBT abundance between the strains in the iron-limiting medium, there was a drastic 11-fold change in the ratio of culture supernatant-associated MBT to cell pellet-associated MBT between the strains, with the mutant having a significantly larger proportion of cell-associated MBT. Overall, the increased abundance of MBT in the mutant is consistent with its OP phenotype. All together, these findings support the hypothesis that the mutant overproduces MBT due to an inability to secure a suitable iron supply in the presence of a malfunctioning ESX-3 with an impaired capacity for utilization of MBT-bound iron in both iron-limiting and iron-rich media.

## 3. Discussion

ESX-3 is essential for *Mtb* and *M. bovis* BCG growth in standard MB growth media, a property of ESX-3 thought to be due to its requirement for utilization of MBT/cMBT-bound iron by a yet unknown mechanism. Notably, the ESX-3 essentiality seen in these slow-growing mycobacteria is not recapitulated in *Mab*, despite the involvement of *Mab* ESX-3 in iron homeostasis, as determined by this study. Our findings demonstrate a functional link between the ESX-3 and siderophore systems of *Mab*. We found that *Mab* Tn mutants with insertions in the *esx-3* locus have an OP phenotype reminiscent of the pigmentation observed in the *Mtb* ESX-3 mutant. Based on our results and by analogy to the observation made for *Mtb*, we attribute the pigmentation of the *Mab* mutants to an abnormally high accumulation of MBT–Fe^3+^ complex. We hypothesize that a dysfunctional ESX-3 in the *Mab* mutants impairs the utilization of MBT-bound iron, a defect that, in turn, leads to a constant intracellular iron-deficiency state accompanied by de-repression of IdeR-regulated MBT biosynthesis genes and excessive accumulation of the MBT–Fe^3+^ complex. Our laboratory hopes to explore the experimental validation of this hypothesized model in the future.

Our results show that the impact of a compromised ESX-3 on the iron uptake capability of the *Mab* mutants is not severe enough to prevent growth under iron-limiting or iron-rich conditions. This finding underlines a difference between the iron-acquisition capabilities of *Mab* and the slow-growing mycobacteria noted above. Paralleling results seen with *Mtb* [45], however, we found that the MBT/cMBT biosynthesis inhibitor salicyl-AMS (which targets MbtA [44,45]) produces a drastic *Mab* growth inhibition conditional to culturing in iron-limiting medium. This finding suggests a critical need of MBT-based iron scavenging and acquisition for *Mab* growth under iron-limiting conditions. Our findings also suggest that the ability of the *Mab* ESX-3 mutants to utilize MBT-bound iron is diminished rather than abolished. There are two possible non-mutually exclusive explanations for the iron acquisition ability of the mutants. They could acquire iron using a partially functional ESX-3 or via a secondary mechanism that allows for utilization of MBT-bound iron in an ESX-3 independent manner. Future *Mab* studies are warranted to probe the hypothesized essentiality of *mbtA* and siderophore production for growth in iron-limiting media, explore the salicyl-AMS’ mechanism of antimicrobial activity, and further investigate bacterial iron acquisition.

Our serendipitous finding that the OP phenotype of the colonies of *Mab* ESX-3 mutants is conditional to the presence of BSA on s7H11 plates and is BSA lot-dependent was unexpected. The mechanism underlying this phenomenon remains unknown. Nevertheless, the observation that the properties of the BSA added to the growth medium can influence the behavior of mycobacterial cultures is not unprecedented [67]. Moreover, a study reporting that addition of BSA to cultures of *Pseudomonas putida* leads to an enhancement of siderophore accumulation in the medium provides another example of a puzzling link between BSA in the medium and siderophore production [68]. The finding that our *Mab* mutants do not display the OP phenotype and have WT-like growth on 7H11 plates lacking BSA suggests that the mutants are able to secure a suitable (non-limiting) iron supply (conceivably via MBT-dependent and/or MBT-independent mechanisms) in the absence of BSA. The addition of BSA to the 7H11 medium appears to compromise the ability of *Mab* to procure a suitable iron supply in the absence of a WT ESX-3. A tantalizing mechanistic possibility behind this phenomenon is that binding of MBT and/or the MBT–Fe^3+^ complex to BSA may exacerbate the malfunctioning of an already weakened iron acquisition system in the mutants. The documented binding of the enterobactin siderophore to BSA and the earlier proposal that serum albumin may act in conjunction with other proteins in serum to restrict the iron supply needed for pathogen growth provide a conceptual framework for this possibility [69].

We expected our screen of a near-saturation library with ~196,000 Tn mutants to provide the first collection of *Mab* mutants with gene knockouts of individual genes in the 14-kb *esx*-*3* locus and, possibly, illuminate any genetic determinants outside the locus that are involved in ESX-3 function or transport of ESX substrates through the mycolate layer [21]. The screen rendered a collection of 54 mutants. Of the 54 mutants, 47 had an insertion in one of nine (out of eleven) genes in the *esx-3* locus, two had an insertion in a hypothesized promoter region at the 5′-end of the locus, and five had an insertion distant from the locus. Interestingly, the screen did not render mutants of *esxG* or *espG3*, two genes for which, given their size, the theoretical probability of not having their Tn mutants in our library by chance is in the order of 1 × 10^−5^ and 1 × 10^−15^, respectively (Figure S3). Thus, our results suggest that disruption of *esxG* or *espG3* does not lead to the OP phenotype. Functional redundancy might be a reason for the lack of *esxG* and *espG3* mutant isolates. *Mab* has two *esxG* paralogues encoding proteins with 98% (*MAB_0048*) and 99% (*MAB_0665*) sequence identity to EsxG (Figure S7a,b). One or both of these paralogues might complement an *esxG* knockout. *Mab* appears to have only one *espG3* paralogue (*MAB_0147c*) encoding an EspG family protein with ~22% amino acid identity to EspG3 (not shown). The low sequence identity between the two proteins is not surprising, as EspG paralogues tend to have low sequence identity (<25%) [70,71]. Perhaps *MAB_0147c* can complement an *espG* knockout to a degree sufficient to prevent the pigmentation phenotype. Interestingly, *MAB_0147c* is preceded by, and possibly in the same operon with, *MAB_0149c* and *MAB_0148c*, which encode a PE protein (17% identity with Pe5, Figure S7a) and a PPE protein (21% identity with Ppe4, Figure S7b), respectively. The *MAB_0149c−MAB_0147c* cluster is not in proximity to either the *esx-3* locus or the *esx-4* locus (*MAB_3759c−MAB_3753c*), the only two *esx* loci in *Mab* [17,72]. Of note, the *MAB_0149c−MAB_0147c* array is homologous with the *pe-ppe-espG* array of the so-called *esx-4*_EVOL_ locus present in *Mab* subsp. *bolletii* and *Nocardia brasiliensis* [17]. The function of the genes in the *MAB_0149c−MAB_0147c* cluster remains unknown.

Our mutant complementation analysis showed weak partial complementation for some mutants with insertions in *esx-3* locus genes. The poor complementation seen for some of these genes could be due to polar effect and/or loss of translational coupling. The latter possibility is likely in the case of the *esxH* mutant, which can be fully complemented by an *esxG*-*esxH* fragment. Interestingly, complementation of the *Mtb esxH* mutant has been reported only with the cognate *esxG*-*esxH* pair [33]. Moreover, folding of the co-expressed *Mtb* EsxG and EsxH proteins is coupled to formation of a stable 1:1 heterodimeric complex [28,73,74], a process likely facilitated by translational coupling. Of the five mutants with insertions outside the *esx-3* locus, only *MAB_2276c* and *MAB_4783* could be complemented, albeit partially. Prediction of operonic arrangements suggests that the weak and lack of complementation of the *MAB_4783* and *MAB_1912c* mutants, respectively, might be caused by polar effects resulting from the Tn insertions (Table S4). Conversely, polar effect is unlikely to be responsible for the weak complementation of the *MAB_2276c* mutant or the lack of complementation of the *MAB_4275c* and *MAB_4537c* mutants (Table S4). Since only one Tn mutant was isolated for each of the five loci outside the *esx-3* gene cluster, it is also possible that spontaneous mutations in the genome (e.g., possibly in the *esx-3* locus) are ultimately responsible for the pigmentation seen in at least some of these mutants. Future experiments to further investigate genotype–phenotype associations in the mutants with weak or no complementation will be needed.

The number of Tn mutant isolates with insertions in the 14-kb *esx-3* locus is lower than expected given the size of the library screened, which corresponds to an average of one Tn insertion per 26 bp. Assuming that no insertion compromises viability and that the probability of insertion is uniform across the genome, one could have predicted approximately 540 insertions in the *esx-3* locus. Thus, it is somewhat surprising that the screen rendered 49 isolates with insertions in the locus (one Tn/286 bp observed average), even considering that not all insertions in the locus could be expected to produce colonies with OP phenotype. For example, some gene knockouts might not lead to the phenotype due to functional redundancies (e.g., *esxG*), some intragenic insertions near a gene’s 3′ end might not effectively disrupt function, and some insertions might be selected against due to generation of toxic protein truncations. A trans-complementation mechanism preventing the OP phenotype on the screen plates might have also contributed to lower the number of isolates. Mutants with an impaired ESX-3 could perhaps utilize ESX-3 substrates involved in acquisition of MBT-bound iron release into the medium by nearby colonies with a functional secretion system. The observation that the growth defect of the *Mtb* ESX-3 mutant can be eliminated by filtered supernatant from a culture of the WT strain or by co-culturing with the WT strain [24] provides some conceptual support for the possibility of a trans-complementation phenomenon in our screen plates.

The Tn insertion distribution pattern in the pool of isolates with insertions in the *esx-3* locus was also unexpected. For example, we found four insertions in *esxH* (291 bp; one Tn/73 bp average) and fifteen insertions in *ppe4* (1572 bp; one Tn/105 bp average), but only six insertions in *eccC3* (4032 bp; one Tn/672 bp average). Interestingly, an irregular insertion pattern across the length of some genes was also evident. Most notably, the seven insertions in *eccA3* (1863 bp) were exclusively distributed across the first half of the gene (~1000-bp segment; one Tn/143 bp local average), and the only three insertions in *eccB3* (1566 bp) mapped to the first third of the gene (545-bp segment; one Tn/182 bp local average). The unexpected distribution of insertions in the locus is unlikely to be due to pure chance. As noted above, selection against insertions leading to production of toxic protein truncations might have contributed to biases in insertion patterns. On the other hand, the insertion pattern in some of the genes might signify the presence of functionally dispensable and functionally indispensable (sub)domains in their protein products. This is a tempting possibility for *eccA3*. EccA3 proteins have an *N*-terminal domain containing tetratricopeptide repeats (postulated to mediate interactions with ESX-3 substrates) and a *C*-terminal ATPase domain [75]. All the *eccA3* mutants identified had insertions distributed across the *N*-terminal domain, perhaps suggesting that disruption of the ATPase domain does not lead to an OP phenotype. Intragenic Tn insertion patterns displaying segments with and segments without insertions that correspond to dispensable and essential protein domains, respectively, have in fact been reported [76,77].

Our structural analysis of the *Mab* siderophore indicates that MBT Ab is an unusual MBT variant. The siderophore has considerable similarity to the recently proposed structure MBT M from *M. marinum*, a slow-growing mycobacterium that causes tuberculosis-like disease in fishes and opportunistic infections in humans, most commonly leading to skin and soft tissue disease. Both MBTs have structural features uncommon in mycobacterial siderophores, but evocative of nocardial MBT-like compounds with cytotoxicity or antiproliferative activity. This finding raises the possibility of roles for MBTs Ab and M in pathogenesis unlinked to iron uptake. MBTs Ab and M lack a characteristic long-chain acyl substituent found on the internal hydroxylysine residue of other MBTs characterized thus far (e.g., *Mtb* and *Msm* MBTs). The lipid tail installed on the hydroxylysine is thought to be critical for the ability of the siderophores to interact with biological membranes and capture iron within macrophages through lipid trafficking [65,78]. The long alkyl chains in the polyketide synthase-derived hydroxy acid moieties of MBT Ab and MBT M (and related nocardial compounds) are likely the functional equivalent of the lipid tail moieties in the MBTs of *Mtb*, *Msm*, and other mycobacteria. It appears that evolution might have led to two different strategies to provide the hydrophobic tail needed for the interactions of the siderophores with lipidic environments of both bacterial and host cells.

## 4. Materials and Methods

### 4.1. Routine Culturing Conditions, Molecular Biology Techniques, and Reagents

Unless otherwise stated, *Mab* (type strain ATCC 19977^T^) and its derivatives were cultured under standard conditions in MB 7H9 broth (Difco, Becton-Dickinson and Co., Franklin Lakes, NJ, USA) supplemented with 10% ADN (5% BSA, 2% dextrose, 0.85% NaCl) and 0.05% Tween 80 (s7H9 broth), or ADN-supplemented MB 7H11 agar (s7H11) (Difco), as reported [43]. Growth curve experiments were carried out using a 96-well plate-based platform as described previously [79]. Where appropriate, hygromycin (Hyg, 1000 μg/mL) and/or zeocin (Zeo, 50 μg/mL) were added to the mycobacterial growth media. *Escherichia coli* strains were cultured under standard conditions in Luria–Bertani media [80]. When required, ampicillin (Amp, 100 μg/mL), kanamycin (Km, 30 μg/mL), Hyg (200 μg/mL), Zeo (50 μg/mL), and/or 5-bromo-4-chloro-3-indolyl-β-d-galactopyranoside (20–30 μg/mL) were added to the Luria–Bertani media. Unless indicated otherwise, DNA manipulations were carried out using established protocols and *E. coli* as the primary cloning host [80]. PCR-generated DNA fragments used in plasmid constructions were sequenced to verify fidelity (Genewiz, Azenta Life Sciences, Chelmsford, MA, USA). *Mab* genomic DNA isolation, plasmid electroporation into *Mab,* and selection of *Mab* transformants were carried out following standard protocols [43,44,81]. Unless otherwise stated, reagents were purchased from Sigma-Aldrich Inc. (St. Louis, MO, USA), Thermo Fisher Scientific Inc. (Branchburg, NJ, USA), New England Biolabs Inc. (Ipswich, MA, USA), Qiagen LLC. (Germantown, MD, USA), or VWR International, LLC (Radnor, PA, USA). Information on oligonucleotides, mycobacterial gene expression plasmids, and mycobacterial strains used in this study is provided in Tables S1–S3, respectively.

### 4.2. Construction of Plasmid pMOD3Zeo and Preparation of EZ-Tn5-Derived Transposome

The EZ-Tn5-carrying plasmid pMOD-3 from the EZ-Tn5 Custom Transposome Construction Kit (Epicentre Biotechnologies Corp., Madison, WI, USA) was modified by introducing a Zeo resistance (Zeo^R^) selectable marker for *Mab* [81]. To this end, the promoter-Zeo^R^ gene (*sh ble*) fragment from plasmid pMSG360Zeo [82] was amplified with primers PM57 and PM56. The latter primer introduced the rrnB-T1 transcription terminator [83] at the 3′ end of the promoter*─*s*h ble* cassette. The resulting amplicon was cloned into vector pCR-2.1-TOPO (TOPO TA Cloning Kit, Thermo Fisher Scientific Inc.). The promoter*─*s*h ble─*terminator cassette insert in the pCR-2.1-TOPO construct was subsequently recovered as a 727-bp KpnI-PstI fragment and subcloned into pMOD-3 digested with KpnI and PstI. The resulting plasmid, named pMOD-3Zeo, carried a Tn5 transposon derivative (hereafter referred to as Tn5Zeo) with a Zeo^R^ marker. Plasmid pMOD-3Zeo was used as a source of Tn5Zeo for the generation of the transposome complex in vitro, following recommended protocols (EZ-Tn5 Transposome Kit, Epicentre Biotechnologies Technologies Corp.).

### 4.3. Generation and Screening of Mutant Libraries

Transposome electroporations into *Mab* were carried out as noted above for plasmids, except for the addition of a glycine treatment during the preparation of electrocompetent cells [84]. Prior to library screening, the number of Tn mutants per milliliter resulting from the electroporations was determined via titration on standard s7H11 plates containing Zeo. The titers obtained guided subsequent bacterial plating for library screening on the same medium or s7H11 with an optimized BSA concentration (s7H11*) as noted in the results section and at a frequency of 100–125 colonies per plate (standard Petri dish size). Plates were incubated at 37 °C and visually screened for colonies with OP phenotype on days 4, 5, and 6. Colonies with OP were recovered from the plates and subjected to colony purification (two rounds of streak plating for colony isolation) and phenotype confirmation. Glycerol stocks (s7H9 containing 25% glycerol) were prepared for each confirmed isolate and stored at −80 °C until needed.

### 4.4. Insertion Site Determination and Southern Blot Hybridization Analysis

Cloning of Tn-containing genomic fragments by plasmid rescue in *E. coli* EC100D-pir+ (Epicentre Bioechnologies Corp.) and Tn-genome junction sequencing were carried out as reported [54], except for the use of Tn5Zeo-specific sequencing primers (primers GB27 and GB28). The genomic sequences identified were mapped onto the *Mab* ATCC 19977^T^ genome (chromosome: GenBank CU458896.1; plasmid pMAB23: GenBank CU458745.1). Southern blot analysis was performed using standard methods described elsewhere [54], except for the use of a Tn5Zeo-specific DNA hybridization probe. The probe was a DIG-labeled DNA fragment (751-bp) generated by PCR amplification (primers GG3 and ME-Plus9-FWD) from pMOD-3Zeo using a PCR digoxigenin-labeled probe synthesis kit (F. Hoffmann-La Roche, Ltd., Nutley, NJ, USA) according to the manufacturer’s instructions.

### 4.5. Construction of Mycobacterial Gene Expression Plasmids

To construct pML1335-Pmyc1tetO-eccB3, a PCR-generated fragment containing *eccB3* (primers MF88 and MF78) and a PCR-generated fragment containing the TetR-responsive promoter Pmyc1tetO (primers MF86 and MF87) from plasmid pSE100 [85] were independently cloned into pCR-2.1-TOPO using *E. coli* TetR (strain DH5α carrying the TetR-expressing plasmid pACBB-TetR-LVA [86]) as the cloning host. The *eccB3* and Pmyc1tetO inserts in the pCR-2.1-TOPO constructs were subsequently recovered as AflII-NotI and BspHI-AflII excerpts, respectively, and the two excerpts were subcloned into the BspHI/NotI-linearized pML1335 vector backbone [87] using *E. coli* TetR as the cloning host. The construction placed *eccB3* under the control of the Pmyc1tetO promoter [85]. To create pML1335-Pmyc1tetO-eccA3, a PCR-generated fragment containing *eccA3* (primers MF79 and MF80) was digested with AflII and SbfI, and then cloned into the pML1335-Pmyc1tetO vector backbone (obtained by AflII-SbfI digestion of pML1335-Pmyc1tetO-eccB3) using *E. coli* TetR as the cloning host. The construction placed *eccA3* under the control of the Pmyc1tetO promoter. To generate plasmids pML1335-Pmyc1tetO-eccC3, -esxH, -mycP3 and -MAB_2276c^+^, four PCR-generated fragments each containing a specific gene (*eccC3*: primers MF75 and MF76; *esxH*: primers MF73 and MF74; *mycP3*: primers KL45 and KL46; and *MAB_2276c*^+^: primers MF81 and MF82) were independently cloned into pCR-2.1-TOPO. Then, the *eccC3*, *esxH*, *mycP3*, and *MAB_2276c*^+^ inserts of the respective pCR-2.1-TOPO constructs were recovered as AflII-NotI fragments and independently cloned into the pML1335-Pmyc1tetO vector backbone (obtained by AflII-NotI digestion of pML1335-Pmyc1tetO-eccB3) using *E. coli* TetR as the cloning host. The constructions placed each *Mab* gene under the control of the Pmyc1tetO promoter. To obtain plasmids pML1335-WCB2-eccD3, -eccE3, -pe5, -ppe4, -esxGH, -MAB_1912c, -MAB_4275c, -MAB_4537c and -MAB_4783, nine PCR-generated fragments each containing a specific gene (*eccD3*: primers MF7 and MF60; *eccE3*: primers MF3b and MF56; *pe5*: primers MF17 and MF62; *ppe4*: primers MF15 and MF51; *esxGH*: primers MF13 and MF50; *MAB_1912c*: primers MF44b and MF54; *MAB_4275c*: primers MF64 and MF65; *MAB_4537c*: primers MF46 and MF55; and *MAB_4783*: primers MF66 and MF67) were independently cloned into pCR-2.1-TOPO. Then, the insert of each pCR-2.1-TOPO construct was recovered as a PsiI-MfeI fragment and independently cloned into the pML1335-WCB2 vector backbone (obtained by PsiI-MfeI digestion of pML1335-WCB2 [54]). The constructions placed each *Mab* gene under the control of the strong constitutive synthetic mycobacterial promoter MOP [88].

### 4.6. Sequence Bioinformatics

Potential IdeR binding sites were identified by querying the *Mab* genome with the IdeR binding site consensus (a.k.a. iron box, TWAGGTWAGSCTWACCTWA; where W = A/T and S = G/C) [57,58]. Potential Zur binding sites were identified by querying selected promoter regions in the chromosome of *Mab* or *Msm* (strain MC2 155; Genbank: NC_008596.1) with the Zur binding site consensus (SNTRWYGAWAAYMRTKKYCRWYADNV; where S = G/C, R = A/G, W = A/T, Y = C/T, M = A/C, K = T/G, D = A/G/T, V = A/C/G, and N = any base) [27,56]. The computational queries were done using the Virtual Footprint tool of the Prokaryotic Database of Gene Regulation (http://www.prodoric.de, accessed on 17 August 2022) [89]. The searches were set to identify sequences with up to five mismatches relative to the consensus, a binding site search criterion used with other mycobacteria [57,60]. Predicted -10 and -35 promoter sequences were identified using the BPROM promoter prediction tool of the Softberry application package (www.softberry.com, accessed on 17 August 2022; Softberry, Inc., Mount Kisco, NY, USA). Potential orthologues of *Mab* genes in *Mtb* (strain H37Rv, Genbank: NC_000962.3), *Msm*, and *M. avium* subsp. *paratuberculosis* (strain K-10, Genbank NC_002944.2) were identified using the orthologue database OrtholugeDB (http://www.pathogenomics.sfu.ca/ortholugedb, accessed on 17 August 2022) [90] or the reciprocal BLAST feature embedded in the GVIEW Server (https://server.gview.ca/, accessed on 17 August 2022) with expected cut-off, alignment length cut-off, and percent identity cut-off of 1 × 10^−10^, 100, and 60, respectively. Routine sequence alignments were performed with Clustal W embedded in the MegAlign module of the DNASTAR Lasergene software package (DNASTAR, Inc., Madison, WI, USA).

### 4.7. Growth Inhibition Assay

Dose−response experiments were carried out using a microdilution assay comparable to those we have reported [44,45,91]. Briefly, mid-log phase cultures in s7H9 medium were spun down and resuspended in the iron-limiting GAST medium or in GAST supplemented with 200 µM FeCl_3_ (GAST+Fe) [44,45,91]. The resulting cell suspensions were used to start multi-well plate microcultures (200 μL/well) at an initial optical density at 595 nm (OD_595_) of 0.001. The MBT/cMBT biosynthesis inhibitor salicyl-AMS (obtained as reported [92]) was evaluated using a 2-fold dilution series covering a 0.004−10,000 μM range. The inhibitor was added from a 10% DMSO stock solution. The final DMSO concentration in inhibitor-treated cultures and DMSO controls (no inhibitor) was 0.5%. Growth was assessed as OD_595_ after four days of incubation (37 °C, 170 rpm) using a DTX 880 multimode detector microplate reader (Beckman Coulter, Inc., Brea, CA, USA). Dose–response data were analyzed with Prism v6.01 (GraphPad Software, Inc., San Diego, CA, USA).

### 4.8. Determination of Mycobactin Production by Spectrophotometric and Radiometirc Assays

Radio-thin layer chromatography (TLC) analysis of radiolabeled MBT was carried out as reported [43,44,45]. Briefly, ^14^C-labeled MBT was obtained by feeding the MBT-specific radiotracer [^14^C]salicylic acid (sp. Act. = 55 µCi/µmol; American Radiolabeled Chemicals, Inc., St. Louis, MO, USA) to cultures in iron-limiting GAST medium, the labeled MBT was extracted from the cultures with organic solvents, and the extracts were subjected to radio-TLC analysis on silica gel plates. Developed TLC plates were exposed to phosphor screens, which were subsequently scanned using a Cyclone Plus Storage Phosphor system (Perkin-Elmer Life and Analytical Sciences, Inc., Boston, MA, USA). Presence of the MBT–Fe^3+^ complex in culture supernatants was evaluated using a spectrophotometric assay measuring the characteristic absorbance of the complex at 450 nm [93]. Absorbance determinations were done using the plate reader noted above.

### 4.9. Mass Spectrometry Analysis of Mycobactins

Cultures of *Mab* WT and the mutant M22^mycP3^ (5 mL, 220 rpm, 50 mL culture tubes) in iron-limiting GASTD medium or in GASTD supplemented with 100 µM FeCl_3_ (GASTD+Fe) [44,45,91] were grown to saturation, normalized to an OD_600_ of 1.4, and centrifuged to obtain cell pellets and spent culture supernatants. MBT siderophores associated with pellets and supernatants were extracted into organic solvents using established protocols reported for isolation of MBT and cMBT siderophores [43,45]. After extraction, the solvent was evaporated and the remaining residue was lyophilized to dryness. The dried siderophore extracts were analyzed by high-resolution liquid chromatography-mass spectrometry (LC-MS). LC-MS was performed on an Agilent 6550 iFunnel Q-TOF mass spectrometer coupled to an Agilent 1290 Infinity LC system (including a binary pump, diode array detector, and autosampler). Data were analyzed using Agilent’s MassHunter Qualitative Software (version B.06.00; Agilent Technologies, Lexington, MA, USA). Chromatography was performed using an Agilent Poroshell 120 SB-C18 column (2.7 μm, 2.1 × 50 mm) at 45 °C and a gradient of solvents A (water, 0.1% formic acid) and B (acetonitrile, 0.1% formic acid) from 5–95% solvent B or 50–100% solvent B in 10 min at a flow rate of 0.4 mL/min. Stock solutions of the siderophore samples were prepared by dissolving the extracts in acetonitrile and routinely stored at −80 °C until needed. Small aliquots of these stocks were diluted 100× and 5 μL of the diluted samples were injected for analysis. The following settings were applied to the electrospray ionization source: gas temperature, 250 °C; nebulizer, 30 psig; sheath gas temperature, 250 °C; vcap, 3500 V; and nozzle voltage, 2000 V. For MS analysis*,* full scan mass spectra (*m*/*z* = 100–1500) were acquired in positive-ion mode. For targeted MS/MS analyses, collision energies (CE) of 10–70 were evaluated. Compounds of interest were identified by both assessing UV chromatograms at 450 nm, and mass spectra exhibiting characteristic Fe isotopic distribution. The commercially available MBT J from *Mycobacterium avium* subsp. *paratuberculosis* [46] (Allied Monitor, Inc., Fayette, MO, USA) was analyzed by MS/MS, and its fragmentation pattern was used as a template to help determine potential structures of the siderophore analogues found in the extracts.

## 5. Conclusions

Overall, our studies provide further insight into the ESX-3 and siderophore systems of *Mab* and expand our knowledge of the biology of this recalcitrant pulmonary pathogen. The findings of this work highlight the need for further research to better understand the functional dimensions of ESX-3 and its interplay with the MBT Ab-mediated iron acquisition system. The collection of novel mutants generated in this study will facilitate progress on these fronts. Mutants generated herein will also be useful to probe the relevance of individual ESX-3 components in cellular and animal infection models. The structural features of the MBT Ab revealed by our study suggest that the siderophore might have cytotoxic properties that contribute to pathogenesis. This possibility warrants exploration and may open new lines of experimental inquiry into the pathogenesis of *Mab*.

## Figures and Tables

**Table 1:** Transposon insertion sites in *M. abscessus* isolates.

Disrupted Gene or Promoter/5′ UTR ^1^	Mutant Isolate ^2^	Insertion Site ^3^	Genome Coordinates ^4^
*eccA3* promoter	M68	ATTCATGGC::Tn::ATTCATGGC	2261555–2261563
	M74	CCCTTCACC::Tn::CCCTTCACC	2261304–2261312
*eccA3*	M20	GCCACATCC::Tn::GCCACATCC	2261245–2261253
	M26	GAGGTAGGAC::Tn::GAGGTAGGAC	2260629–2260637
	M49	GTTCCAGACC::Tn::GTTCCAGACC	2260292–2260300
	M54	GCCCAGGGT::Tn::GCCCAGGGT	2260677–2260685
	M65	ACTCGAGGT::Tn::ACTCGAGGT	2260934–2260942
	M73	GCCTCGGCC::Tn::GCCTCGGCC	2260327–2260335
	M86	GTCGTGGGG::Tn::GTCGTGGGG	2260705–2260713
*eccB3*	M6	GCCTCGCAC::Tn::GCCTCGCAC	2258894–2258902
	M59	GGCCTGCAC::Tn::GGCCTGCAC	2258929–2258937
	M76	GAGCACCGG::Tn::GAGCACCGG	2259061–2259069
*eccC3*	M1	GATCAATACC::Tn::GATCAATACC	2257252–2257260
	M23	CAGCTGGGA::Tn::CAGCTGGGA	2256557–2256565
	M52	CGCCAGCGG::Tn::CGCCAGCGG	2255501–2255509
	M58	ATGTTGGGGG::Tn::ATGTTGGGGG	2254359–2254367
	M79	GCCCTGCAC::Tn::GCCCTGCAC	2256146–2256154
	M80	GGCCTCGGC::Tn::GGCCTCGGC	2254127–2254135
*eccD3*	M75	GCGCCAGGC::Tn::GCGCCAGGC	2250160–2250168
	M77	GATCAGACC::Tn::GATCAGACC	2249549–2249557
*eccE3*	M5	GGCCAGCAT::Tn::GGCCAGCAT	2247334–2247342
	M41	TGCCAACGG::Tn::TGCCAACGG	2246719–2246727
	M62	GGCTTGTCC::Tn::GGCTTGTCC	2246741–2246749
*mycP3*	M22	GTCGCGCAC::Tn::GTCGCGCAC	2248622–2248630
	M44	ACTCAGTGC::Tn::ACTCAGTGC	2247566–2247574
	M53	GCGCAACGC::Tn::GCGCAACGC	2248266–2248274
	M57	GGGTTTGAC::Tn::GGGTTTGAC	2248632–2248640
	M61	GCGCAACGC::Tn::GCGCAACGC	2248266–2248274
	M69	GGCATACAC::Tn::GGCATACAC	2247626–2247634
	M78	GACCAATTC::Tn::GACCAATTC	2248147–2248155
*esxH*	M2	AGTTGAAAG::Tn::AGTTGAAAG	2251565–2251573
	M70	CACCGGCGT::Tn::CACCGGCGT	2251538–2251546
	M72	GGTCAGCAC::Tn::GGTCAGCAC	2251493–2251501
	M84	GCCAGGCGG::Tn::GCCAGGCGG	2251424–2251432
*ppe4*	M7	GTCCAGCCC::Tn::GTCCAGCCC	2252181–2252189
	M45	CTGCTGGGT::Tn::CTGCTGGGT	2253289–2253297
	M47	GGCCAGTCC::Tn::GGCCAGTCC	2251991–2251999
	M51	CTCCTGCAC::Tn::CTCCTGCAC	2252431–2252439
	M60	CTCCTGCAC::Tn::CTCCTGCAC	2252431–2252439
	M63	CTCCTGCAC::Tn::CTCCTGCAC	2252431–2252439
	M66	TCTCCGAGG::Tn::TCTCCGAGG	2252097–2252105
	M67	AAGCCAAGC::Tn::AAGCCAAGC	2252171–2252179
	M71	CAGCAGCGC::Tn::CAGCAGCGC	2253481–2253489
	M81	CCTCGTATG::Tn::CCTCGTATG	2252754–2252762
	M82	CCACCAAGA::Tn::CCACCAAGA	2252676–2252684
	M85	GGCGAAGCC::Tn::GGCGAAGCC	2252167–2252175
	M87	GGGCGGCCA::Tn::GGGCGGCCA	2252946–2252954
	M88	TGCCGAGGC::Tn::TGCCGAGGC	2252329–2252337
*pe5*	M64	GCCCAGCTC::Tn::GCCCAGCTC	2253630–2253638
			
*MAB_4275c* ^5^	P5	GTAGCCGAA::Tn::GTAGCCGAA	4348013–4348021
*MAB_2276c*	M50	GAGCATGCGC::Tn::GAGCATGCGC	2324048–2324056
*MAB_4537c*promoter	M55	CAAGGAAAT::Tn::CAAGGAAAT	4620490–4620498
*MAB_1912c*	M56	TCCAGGACC::Tn::TCCAGGACC	1909545–1909553
*MAB_4783*	M83	AGCGCATGT::Tn::AGCGCATGT	4893827–4893835

^1^ UTR, untranslated region. ^2^ The position and orientation of the transposon in each *esx-3* locus mutant are displayed in Figure 3. ^3^ The direct repeats resulting from the most common 9-bp or the less frequent 10-bp duplication [53] at the transposon insertion point are depicted. ^4^ The 5′ and 3′ chromosomal coordinates of the duplicated segment at the insertion site are noted on the right column. ^5^ The genes below the gray-colored row are outside the *esx-3* locus.

## Data Availability

Not applicable.

## Supplementary Materials

The following supporting information can be downloaded at: https://www.mdpi.com/article/10.3390/pathogens11090953/s1, Figure S1: Southern blot analysis of *M. abscessus* mutants; Figure S2: Effect of BSA on the development of the orange pigmentation phenotype in *M. abscessus* mutants; Figure S3: Probability (*p*) of missing a gene with “x” base pairs (bp) in a library of “n” transposon (Tn) mutants of *M. abscessus*; Figure S4: Complementation of the *esxH* mutant; Figure S5: The orange pigmentation phenotype of *M. abscessus* cultures is influenced by iron availability; Figure S6: Growth of *M. abscessus* strains in iron-rich and iron-limiting media; Figure S7: Substrate candidates for the secretome of the ESX systems of *M. abscessus*; Figure S8: Representative structures and fragmentation patterns of mycobactin Ab (a) and mycobactin J (b); Table S1: Primers used in the study; Table S2: Mycobacterial gene expression plasmids used in this study; Table S3: *M. abscessus* strains included in phenotypic characterizations; Table S4: Genetic loci outside the *esx-3* cluster with transposon insertions; Table S5: Ion peak integration data collected from extracted ion chromatograms for seven different MBT Ab structural variants in samples of supernatant-associated (SA) and cell pellet-associated (CPA) siderophore extracts from cultures in the iron-limiting or iron-rich growth media.
