# Supplementary material for: Mycobacterium abscessus Mutants with a Compromised Functional Link between the Type VII ESX-3 System and an Iron Uptake Mechanism Reliant on an Unusual Mycobactin Siderophore
Source: Pathogens. 2022 Aug 23;11(9):953. doi: 10.3390/pathogens11090953 (PMC9505556; doi:10.3390/pathogens11090953)
Supplement: Supplementary file 1 [file pathogens-11-00953-s001.zip › Supplementary figures captions.pdf]

Supplementary captions

Figure S1. Southern blot analysis of *M. abscessus* mutants. The assemblage of southern blot images shown includes results for thirteen representative mutants of the fifty-four mutants identified in the screen. The set displayed includes nine mutants with insertions in nine *esx-3* genes (mutants used in the complementation analysis, Figure 4), two mutants in the promoter of *eccA3* (Figure 5), and two mutants with intragenic insertions outside the *esx-3* locus (mutants used in complementation analysis, Figure 4). Lane WT, wild-type genomic control digest. Lane M, molecular weight markers. bp, base pairs. Arrows mark positions of observed hybridization bands. Observed fragment sizes are in agreement with expected sizes (*i.e.*, genomic SacII fragment size + Tn size).

Figure S2. Effect of BSA on the development of the orange pigmentation phenotype in *M. abscessus* mutants. (**a**) Representative results showing the need of BSA in the ADN supplement added to the iron-rich 7H11 medium for the development of OP phenotype in *M. abscessus* ESX-3 mutants. Images of streak plates (top) and macrocolonies derived from spot inoculation (bottom). (**b**) The concentration of BSA in the ADN supplement needed for OP development in the *M. abscessus* ESX-3 mutants is BSA-lot dependent. The supplementation modalities assessed did not affect the coloration of the wild-type strain (WT). ADN, standard ADN supplement [43]. DN, ADN with BSA omitted. Conditions leading to OP phenotype are marked with a plus sign (+). A scale bar is shown for the macrocolony images. Petri dishes are standard diameter (100 mm). Images of streak plates and macrocolonies were digitally captured using a T2i DSLR camera (Canon Inc.) after 7 and 5 days of incubation, respectively.

Figure S3. Probability (*p*) of missing a gene with "x" base pairs (bp) in a library of "n" transposon (Tn) mutants of *M. abscessus*. Assuming that the probability of a Tn insertion is uniform across the genome and independent across mutants, the chance of no Tn insertion in a given gene is a negative exponential function of the library size ($p = e^{-x*n/g}$, where x = gene length in bp, n = number of Tn mutants in the library, and g = genome size in bp). In our screened library of 196,000 mutants, the theoretical probability of the Tn missing the reference ~1,000-bp average size gene of *M. abscessus* by chance is $\sim 1 \times 10^{-16}$.

Figure S4. Complementation of the *esxH* mutant. Single colonies (top row) and spot inoculation-derived macrocolonies (bottom row) of the *esxH* mutant (column 1) and the mutant genetically complemented by expression of *esxH* alone (column 2) or by expression of the *esxH-esxG* gene pair (column 3) from pML1335 vector derivatives. Expression of *esxH* alone leads to a slight reduction in pigmentation intensity (weak partial phenotypic complementation), whereas expression of the *esxH-esxG* pair leads to the typical off-white wild-type phenotype (full phenotypic complementation). Scale bar (bottom left corner), 2 mm.

Figure S5. The orange pigmentation phenotype of *M. abscessus* cultures is influenced by iron availability. (**a**) Cultures (C), spent supernatants (S), and pellets (P) of strains grown to saturation in iron-limiting GAST

broth (left panel) or iron-rich GAST+Fe broth (GAST supplemented with $FeCl_3$ to 100 µM; right panel). After incubation for growth, cultures were treated by addition of $FeCl_3$ (to 5 mM; right half of each panel) to allow maximal conversion of the colorless MBT in the cultures into the orange MBT-Fe^{3+} complex, or left untreated (left half of each panel). S-GAST and S-GAST+Fe, sterile GAST and GAST+Fe broth controls, respectively. The sterile broth control tubes and WT culture control tubes shown are the same shown in Figure 7. Data shown in both figures are derived from the same experiments. (**b**) Spectrophotometric quantification of MBT-Fe^{3+} complex in culture supernatants of strains grown to saturation in iron-limiting GAST broth, and then treated by addition of $FeCl_3$ (to 5 mM; red bars) or left untreated (gray bars). A_{450}, blank (sterile broth)-corrected absorbance at 450 nm. The data represent means ± SE of three cultures. The wild-type strain (WT) and $M57^{mycP3}$ carried pML1335 (empty), the vector used in the genetic complementation experiments, so that the strains could be grown along with the complemented strain $M57^{mycP3}$-C in the same antibiotic-containing medium.

Figure S6. Growth of *M. abscessus* strains in iron-rich and iron-limiting media. (**a**) iron-rich standard s7H9 medium, (**b**) iron-limiting GAST medium, and (**c**) iron-rich GAST+Fe (100 µM $FeCl_3$) medium. The growth curve experiments were carried out using a 96-well plate-based platform. Strains were inoculated at an optical density (OD) at 595 nm of 0.005. The wild-type strain (WT) and the mutants carried pML1335 (empty), the vector used in the genetic complementation experiments, so that the strains could be grown in the same antibiotic-containing medium used for the complementation control strains. Data represent means and standard errors of three independent experiments, each with up to six replicate wells per strain.

Figure S7. Substrate candidates for the secretome of the ESX systems of *M. abscessus*. (**a**) Heatmaps showing pairwise sequence identity percentages for predicted Ppe protein paralogues (small identity matrix) and for predicted Pe (names in red) and Esx (names in blue) protein paralogues (large identity matrix). Proteins are labeled with numbers corresponding to those in their cognate *MAB_* locus tags. The alternative protein name of each ESX-3 and ESX-4 canonical (*esx*-locus encoded) substrate is noted. ESX-3 canonical substrates are highlighted in yellow boxes. The number in parentheses next to each protein name on the top listing indicates the number of amino acid residues in the protein. The numbers in parentheses next to each protein name on the right listing denote codes for protein family and domain information from the Pfam (PF), InterPro (IPR), and Superfamily (SSF) protein and domain databases, supporting the depicted protein classification. Protein family/domain code: 1 = PF00934, PE; 2 = PF00823, PPE; 3 = PF18878, PPE-PPW; 4 = PF08237, PE-PPE; 5 = PF06013, WXG100; 6 = PF10824, T7SS_ESX_EspC; 7 = IPR000084, PE-PGRS_N; 8 = IPR036689, ESAT-6-like_sf; 9 = IPR043641, PPE-PPW_C; 10 = IPR000030, PPE_family; 11 =IPR038332, PPE_sf; 12 = IPR013228, PE-PPE_C; 13 = IPR010310, T7SS_ESAT-6-like; 14 = IPR029058, α/β-hydrolase; 15 = IPR022536, EspC; 16 = SSF140453, EsxAB dimer-like superfamily; 17=SSF53474, α/β-Hydrolases superfamily; 18 = IPR000675, cutinase/acetylxylan esterase. The information was mined from the UniProt Knowledgebase database (UniProtKB; https://www.uniprot.org/). Sequence alignment was done with the Clustal W algorithm embedded in the MegAlign application DNASTAR Lasergene software (DNASTAR, Inc.). (**b**) Gene clusters encoding highly conserved paralogues of the ESX-3 substrates Pe5, Ppe4, EsxG, and EsxH. Genes are depicted as arrows and labeled with gene names (*esx-3* locus genes) or numbers corresponding to those in their cognate *MAB_* locus tags as per the genome annotation. Names are colored

following the pattern utilized in (**a**). The amino acid sequence identity between the ESX-3 canonical substrates and their protein paralogues is indicated as per information shown in (**a**). The genes *MAB_0667* and *MAB_0668c* (dark gray genes, bottom cluster) encode proteins unrelated to ESX systems or their substrates.

Figure S8. Representative structures and fragmentation patterns of mycobactin Ab (**a**) and mycobactin J (**b**). The variable R group of mycobactin Ab is highlighted in red.
