# Supplementary material for: Mycobacterium abscessus Mutants with a Compromised Functional Link between the Type VII ESX-3 System and an Iron Uptake Mechanism Reliant on an Unusual Mycobactin Siderophore
Source: Pathogens. 2022 Aug 23;11(9):953. doi: 10.3390/pathogens11090953 (PMC9505556; doi:10.3390/pathogens11090953)
Supplement: Supplementary file 1 [file pathogens-11-00953-s001.zip › Supplementary Tables.pdf]

Supplementary Tables

Table S1. Primers used in the study.

Primer name	Sequence (5′→3′)	Features	Template target (size)
PM57	GCCAATGCATAGACCCCAGGCTTGACACTTTATGCTT	NsiI site	Promoter-*sh ble* (435 bp)
PM56	TGGCATTTAAATAAAACGAAAGGCCCAGTCTTTCGACTGAGCCTTTCATTATACGAAGTTATAAGCTGGGGCGT	SwaI site, rrnB-T1 terminator	
MF79	AAAATTTCATGAAGACCCCAGGCTTGACACTTTATGCTTCCGGCTCGTATAATGTGTGGAATTGTGAGCGCTCACAATTCGCTTAAGGAGGAAAGCTGTAAATGGATATTCGTGAACGA	BspHI and AflII sites, synthetic mycobacterial constitutively promoter (MOP), mycobacterial optimized ribosome binding site (RBS^{op})	*eccA3* (1863 bp)
MF80	GCTCGGCCTGCAGGTTGTTGTCTCGATTGTCGCT	SbfI site	
MF88	TTCGGCCTTAAGGAAGGAGATATACATATGAGCGACAATCGAGACAACAACGCG	AflII site, RBS^{op}	*eccB3* (1566 bp)
MF78	CGTCGAGCGGCCGCCCTGCAGGGACGGCTCAACGGGACTCCT	NotI and SbfI sites	
MF75	CGATGCTCATGAAGACCCCAGGCTTGACACTTTATGCTTCCGGCTCGTATAATGTGTGGAATTGTGAGCGCTCACAATTCGCTTAAGGAGGAAATCCCGATGAGCCGTC	BspHI and AflII sites, MOP, RBS^{op}	*eccC3* (4032 bp)
MF76	CGGTCAGCGGCCGCCCTGCAGGGTTGAGATTCATCGTGGGT	NotI and SbfI sites	
MF7	CCCCTATTATAAGAGTCAGAGGAAACACCATGTCTGACAACGCCG	PsiI site, RBS^{op}	*eccD3* (1389 bp)

MF60	TCGCGGCAATTGTGTGTCATCGGTTGAGGA	MfeI site	
MF3b	CGAGTATTATAATCACGTGAGGAAAACCACCCATGCCTGGAACGC	PsiI site, RBS^{op}	*eccE3* (954 bp)
MF56	GCCGTCCAATTGGCGCTGGCCATCAGACCA	MfeI site	
KL45	ACATGTTCATGAAGACCCCAGGCTTGACACTTTATGCTTCCGGCTCGTATAATGTGTGGAATTGTGAGCGCTCACAATTCGCTTAAGGAGGAAACAACCGATGACACATTTTATCCGCGAT	PciI, BspHI, and AflII sites, MOP, RBS^{op}	*mycP3* (1386 bp)
KL46	ACATGTGCGGCCGCCCTGCAGGAACGTGAACTTCTTACTCGCATGGGTAC	PciI, NotI, and SbfI sites	
MF73	AGGTCATCATGAAGACCCCAGGCTTGACACTTTATGCTTCCGGCTCGTATAATGTGTGGAATTGTGAGCGCTCACAATTCGCTTAAGGAGGAAAGAGAAATGTCGCAGATTACTTTCAACT	BspHI snf AflII sites, MOP, RBS^{op}	*esxH* (291 bp)
MF74	CATGAGGCGGCCGCGCGTTTATGCGCCCCACTTGGCT	NotI site	
MF15	GGCGTATTATAACGCGCGGAGGAAACACCGATGTCGGCTCCGATT	PsiI site, RBS^{op}	*ppe4* (1572 bp)
MF51	GTATTGCAATTGACTTCTGTGGCGGATCAGGT	MfeI site	
MF17	GAGCATTTATAACAGGGAGAGGAAAACCCACGATGAATCTCAACG	PsiI site, RBS^{op}	*pe5* (309 bp)
MF62	CCCGGGCAATTGGCTCAGCAGCGCCGAGTGGA	MfeI site	
MF13	GACGAATTATAAGAGAATGAGGAAAGCATCTTATGAGTTTGCTTGACGCA	PsiI site, RBS^{op}	*esxGH* (607 bp)
MF50	TGAGCACAATTGGCGTTTATGCGCCCCACTTGGCT	MfeI siteon	

MF44b	GCGGCCTTATAACTCGTGGAGGAAACATCTCCATGACGACAACGG	PsiI site, RBS^{op}	*MAB_1912c* (381 bp)
MF54	CTGTCGCAATTGGTCAATAGGTGCCCTCGGGA	MfeI site	
MF81	GTGTGGTCATGAAGACCCCAGGCTTGACACTTTATGCTTCCGGCTCGTATAATGTGTGGAATTGTGAGCGCTCACAATTCGCTTAAGGAGGAAACTTATCGATGCACGCCAAA	BspHI and AflII sites, MOP, RBS^{op}	*MAB_2276c*$^{+}$ (753 bp)
MF82	AGACGGGCGGCCGCCCTGCAGGGTTACACCTGCTCAGGCC	NotI and SbfI sites	
MF64	GGCAGTTTATAAGATACCGAGGAAACGACATGATTGATACCGCAGC	PsiI site, RBS^{op}	*MAB_4275c* (852 bp)
MF65	CGAACTCAATTGCCGCGATGATTCAGCGGCCTTTACGA	MfeI site	
MF46	CTGGATTTATAATGTCTAGAGGAAAGAGCATGCTTGTCTTAACTCGTTGG	PsiI site, RBS^{op}	*MAB_4537c* (1002 bp)
MF55	GGCCGGCAATTGTCTCGTCTTGCTGGAACGCT	MfeI site	
MF66	ATCGGTTTATAAGGCGATGAGGAAAACCCATCATGACAGCTCCGG	PsiI site, RBS^{op}	*MAB_4783* (1491 bp)
MF67	CCCATTCAATTGGGTTCAAAACATCAGCGATCATCA	MfeI site	
MF86	CTGTTTTCATGACTAGAAATATTGGATCGTCGGCACCGTCACG	BspHI site	P_{myc1}*tetO* (296 bp)
MF87	GGGGATGCGGCCGCATCAGCTGTTCTTAAGGCATGCGGATCGTGCTCATTTCGGGC	NotI and AflII sites	
GG3	CAGGCTTGACACTTTATGCTTC	Tn5Zeo-specific primer for hybridization probe generation	Tn5Zeo probe (751 bp)
ME-Plus9-FWD	CTGTCTCTTATACACATCTCAACCATCA	Tn5Zeo-specific primer for hybridization probe generation	

GB27	GCACACTGGCGGCCGTTACTAGTG GATC	Tn5Zeo-specific sequencing primer	Tn-genome junction sequence
GB28	GTACTATCAACAGGTTGAACTGCC	Tn5Zeo-specific sequencing primer	Tn-genome junction sequence

Table S2. Mycobacterial gene expression plasmids used in this study.

**Plasmid**	**Characteristics**	**Source**
pML1335	Mycobacterial L5 integrative vector; backbone used for mycobacterial gene expression; *colE1*, L5 *int*, L5 *attP*, $xylE_m$, *hyg*, p_{smyc}-gfp_m^{2+}	[87]
pML1335ΔxylE	pML1335 derivative lacking the *xylE* marker and used as empty vector control	[54]
pML1335-WCB2	pML1335 derivative used as source of vector backbone for mycobacterial gene expression under control of the MOP promoter	[54]
pML1335-Pmyc1tetO-eccA3	pML1335 derivative expressing *eccA3* under control of the Pmyc1tetO promoter	This study
pML1335-Pmyc1tetO-eccB3	pML1335 derivative expressing *eccB3* under control of the Pmyc1tetO promoter	This study
pML1335-Pmyc1tetO-eccC3	pML1335 derivative expressing *eccC3* under control of the Pmyc1tetO promoter	This study
pML1335-WCB2-eccD3	pML1335-WCB2 derivative expressing *eccD3* under control of the MOP promoter	This study
pML1335-WCB2-eccE3	pML1335-WCB2 derivative expressing *eccE3* under control of the MOP promoter	This study
pML1335-Pmyc1tetO-mycP3	pML1335 derivative expressing *mycP3* under control of the Pmyc1tetO promoter	This study
pML1335-Pmyc1tetO-esxH	pML1335 derivative expressing *esxH* under control of the Pmyc1tetO promoter	This study
pML1335-WCB2-esxGH	pML1335 derivative expressing the *esxG-esxH* gene pair under control of the MOP promoter	This study
pML1335-WCB2-ppe4	pML1335-WCB2 derivative expressing *ppe4* under control of the MOP promoter	This study
pML1335-WCB2-pe5	pML1335-WCB2 derivative expressing *pe5* under control of the MOP promoter	This study

pML1335-WCB2-1912c	pML1335-WCB2 derivative expressing *MAB_1912c* under control of the MOP promoter	This study
pML1335-Pmyc1tetO-2276c$^+$	pML1335 derivative expressing *MAB_2276c*$^+$ under control of the Pmyc1tetO promoter	This study
pML1335-WCB2-4537c	pML1335-WCB2 derivative expressing *MAB_4537c* under control of the MOP promoter	This study
pML1335-WCB2-4275c	pML1335-WCB2 derivative expressing *MAB_4275c* under control of the MOP promoter	This study
pML1335-WCB2-4783	pML1335-WCB2 derivative expressing *MAB_4783* under control of the MOP promoter	This study

Table S3. *M. abscessus* strains included in phenotypic characterizations.

Strain	Characteristics	Source
Wild-type	Type strain ATCC 19977	American Type Culture Collection
$M65^{eccA3}$	Mutant with a Tn insertion in *eccA3* (see Table 1)	This study
$M65^{eccA3}$-C	$M65^{eccA3}$ carrying complementation plasmid pML1335-Pmyc1tetO-eccA3 integrated at the phage L5 *attB* integration site	This study
$M59^{eccB3}$	Mutant with a Tn insertion in *eccB3* (see Table 1)	This study
$M59^{eccB3}$-C	$M59^{eccB3}$ carrying complementation plasmid pML1335-Pmyc1tetO-eccB3 integrated at the phage L5 *attB* integration site	This study
$M23^{eccC3}$	Mutant with a Tn insertion in *eccC3* (see Table 1)	This study
$M23^{eccC3}$-C	$M23^{eccC3}$ carrying complementation plasmid pML1335-Pmyc1tetO-eccC3 integrated at the phage L5 *attB* integration site	This study
$M75^{eccD3}$	Mutant with a Tn insertion in *eccD3* (see Table 1)	This study
$M75^{eccD3}$-C	$M75^{eccD3}$ carrying complementation plasmid pML1335-WCB2-eccD3 integrated at the phage L5 *attB* integration site	This study
$M5^{eccE3}$	Mutant with a Tn insertion in *eccE3* (see Table 1)	This study

M5^{eccE3}-C	M5^{eccE3} carrying complementation plasmid pML1335-WCB2-eccE3 integrated at the phage L5 *attB* integration site	This study
M57^{mycP3}	Mutant with a Tn insertion in *mycP3* (see Table 1)	This study
M57^{mycP3}-C	M57^{mycP3} carrying complementation plasmid pML1335-Pmyc1tetO-mycP3 integrated at the phage L5 *attB* integration site	This study
M72^{esxH}	Mutant with a Tn insertion in *esxH* (see Table 1)	This study
M72^{esxH}-C	M72^{esxH} carrying complementation plasmid pML1335-Pmyc1tetO-esxH integrated at the phage L5 *attB* integration site	This study
M72^{esxGH}-C	M72^{esxH} carrying complementation plasmid pML1335-WCB2-esxGH integrated at the phage L5 *attB* integration site	This study
M45^{ppe4}	Mutant with a Tn insertion in *ppe4* (see Table 1)	This study
M45^{ppe4}-C	M45*ppe4* carrying complementation plasmid pML1335-WCB2-ppe4 integrated at the phage L5 *attB* integration site	This study
M64^{pe5}	Mutant with a Tn insertion in *pe5* (see Table 1)	This study
M64^{pe5}-C	M64^{pe5} carrying complementation plasmid pML1335-WCB2-pe5 integrated at the phage L5 *attB* integration site	This study
M56^{MAB_1912c}	Mutant with a Tn insertion in *MAB_1912c* (see Table 1)	This study
M56^{MAB_1912c}-C	M56^{MAB_1912c} carrying complementation plasmid pML1335-WCB2-1912c	This study

	integrated at the phage L5 *attB* integration site	
$M50^{MAB\_2276c}$	Mutant with a Tn insertion in *MAB_2276c* (see Table 1)	This study
$M50^{MAB\_2276c}$-C	$M50^{MAB\_2276c}$ carrying complementation plasmid pML-MAB_2276c integrated at the phage L5 *attB* integration site	This study
$P5^{MAB\_4275c}$	Mutant with a Tn insertion in *MAB_4275c* (see Table 1)	This study
$P5^{MAB_4275}$c-C	P5MAB_4275c carrying complementation plasmid pML1335-WCB2-4275c integrated at the phage L5 attB integration site	This study
$M83^{MAB\_4783}$	Mutant with a Tn insertion in *MAB_4783* (see Table 1)	This study
$M83^{MAB\_4783}$-C	$M83^{MAB\_4783}$ carrying complementation plasmid pML1335-WCB2-4783 integrated at the phage L5 *attB* integration site	This study

Table S4. Genetic loci outside the *esx-3* cluster with transposon insertions.

Gene name	Protein Information [1]	Potential operon arrangement	Predicted close orthologues
MAB_1912c	Conserved protein, unknown function, glyoxalase-like domain (Pfam: PF18029)	Polycistronic transcription unit: *MAB_1912c* - *MAB_1908*	Orthologues in a limited number of *Mycobacterium* spp.<br>• MYCMA_10010 (100%); *Mycobacteroides abscessus subsp. massiliense*<br>• MASS_1900 (99%); *Mycobacteroides abscessus subsp. bolletii*<br>• BAB75_10435 (94%); *Mycobacteroides immunogenum*<br>• DSM43276_01708 (88%); *Mycobacteroides salmoniphilu*m<br>• BB28_09965 (87%); *Mycobacteroides chelonae*<br>• MSTE_0185 (87%); *Mycobacterium stephanolepidis*<br>• MYCSP_08300 (86%); *Mycobacteroides saopaulen*se<br>• F5544_03720 VO (55%); *Nocardia arthritidis*<br>• DMB37_31995 (54%); *Nocardia sp.* CS682
MAB_2276c	Conserved putative regulatory protein, unknown function, transcriptional activator domain (Pfan: BTAD, PF03704)	Monocistronic transcription unit	Orthologues in a limited number of *Mycobacterium* spp.<br>• MYCMA_09390 (99%); *Mycobacteroides abscessus* subsp. *massiliense*<br>• MASS_2200 (99%); *Mycobacteroides abscessus* subsp. *bolletii*<br>• BAB75_12160 (93%); *Mycobacteroides immunogenum*<br>• MSTE_02489 (63%); *Mycobacterium stephanolepidis*<br>Our cross-species sequence analysis suggests MAB_2276c starts at an ATG codon located 360 bp upstream of the annotated start codon, thus changing the ORF from 130 amino acids to 250 amino acids.

MAB_4275c	Conserved protein, unknown function, DsbD domain (Pfam: PF02683)	Bicistronic transcription unit: *MAB_4276c* - *MAB_4275*	Orthologues in many *Mycobacterium* spp. and beyond
MAB_4783	Conserved PPE family protein; unknown function; PPE domain (Pfam: PF00823)	Bicistronic transcription unit: *MAB_4783* - *MAB_4784*	Orthologues in a limited number of *Mycobacterium* spp.<br>• MASS_4855 (98%); *Mycobacteroides abscessus* subsp. *bolletii*<br>• MYCMA_2621 (98%); *Mycobacteroides abscessus* subsp. *massiliense*<br>• MYCSP_22285 (81%); *Mycobacteroides saopaulense*<br>• BAB75_27080 (81%); *Mycobacteroides immunogenum*<br>• DSM43276_04609 (80%); *Mycobacteroides salmoniphilum*<br>• MSTE_04937 (79%); *Mycobacterium stephanolepidis*<br>• BB28_24355 (79%); *Mycobacteroides chelonae*
MAB_4537c (promoter)	Conserved hypothetical protein, lipoprotein; L,D-transpeptidase catalytic domain (PF17964 and PF03734)	Monocistronic transcription unit	Orthologues in many *Mycobacterium* spp. (*BMC Microbiology* volume 14, Article number: 75, 2014) and beyond.

[1], Protein, operon, and orthology information was mined from the UniProt Knowledgebase database (UniProtKB; https://www.uniprot.org/), the OperonDB database (http://operondb.ccb.jhu.edu/cgi-bin/operons.cgi), and the KEGG GENES Database (https://www.kegg.jp/). The number in parentheses following the gene names on the orthology column indicates the percentage of amino acid identity with the *M. abscessus* protein. The percentage of amino acid identity cut-off for close orthologue designation is >50%.

Table S5. Ion peak integration data collected from extracted ion chromatograms for seven different MBT Ab structural variants in samples of supernatant-associated (SA) and cell pellet-associated (CPA) siderophore extracts from cultures in the iron-limiting or iron-rich growth media.

MBT Ab variant[1] (m/z)	Wild-type strain				*mycP3*::Tn mutant			
	Iron-rich medium		Iron-limiting medium		Iron-rich medium		Iron-limiting medium	
	SA[2]	CPA	SA	CPA	SA	CPA	SA	CPA
813.3606	2,548[3]	9,570	179,680	14,567	73,839	127,555	1,479,897	617,223
841.3919	3,488	6,625	280,196	14,865	111,238	115,465	1,718,215	646,326
855.4075	11,750	15,631	794,105	39,498	296,875	256,437	3,216,418	1,471,093
867.4075	21,410	28,667	1,503,735	76,283	188,928	303,526	2,582,094	911,715
869.4232	59,691	83,166	4,307,673	220,450	1,572,025	1,195,770	338,107	4,883,770
895.4394	30,344	41,162	2,752,619	132,990	357,044	499,650	4,923,891	1,632,656
897.4545	15,777	23,361	1,025,537	56,862	511,399	376,227	6,229,966	1,835,331
**Aggregated data**	145,008	208,182	10,843,545	555,515	3,111,348	2,874,630	20,488,588	11,998,114

[1]: The MBT variant listed are those shown in Figure S8. The values are calculated m/z.
[2]: SA, supernatant-associated siderophore extracts; CPA, cell pellet-associated siderophore extracts.
[3]: All ion peak integration values are averages of two determinations. The data are normalized to 1 ml of cultures with an OD_{600} of 1.0.
