# Supplementary figures and images for: Mycobacterium abscessus Mutants with a Compromised Functional Link between the Type VII ESX-3 System and an Iron Uptake Mechanism Reliant on an Unusual Mycobactin Siderophore
Source: Pathogens. 2022 Aug 23;11(9):953. doi: 10.3390/pathogens11090953 (PMC9505556; doi:10.3390/pathogens11090953)

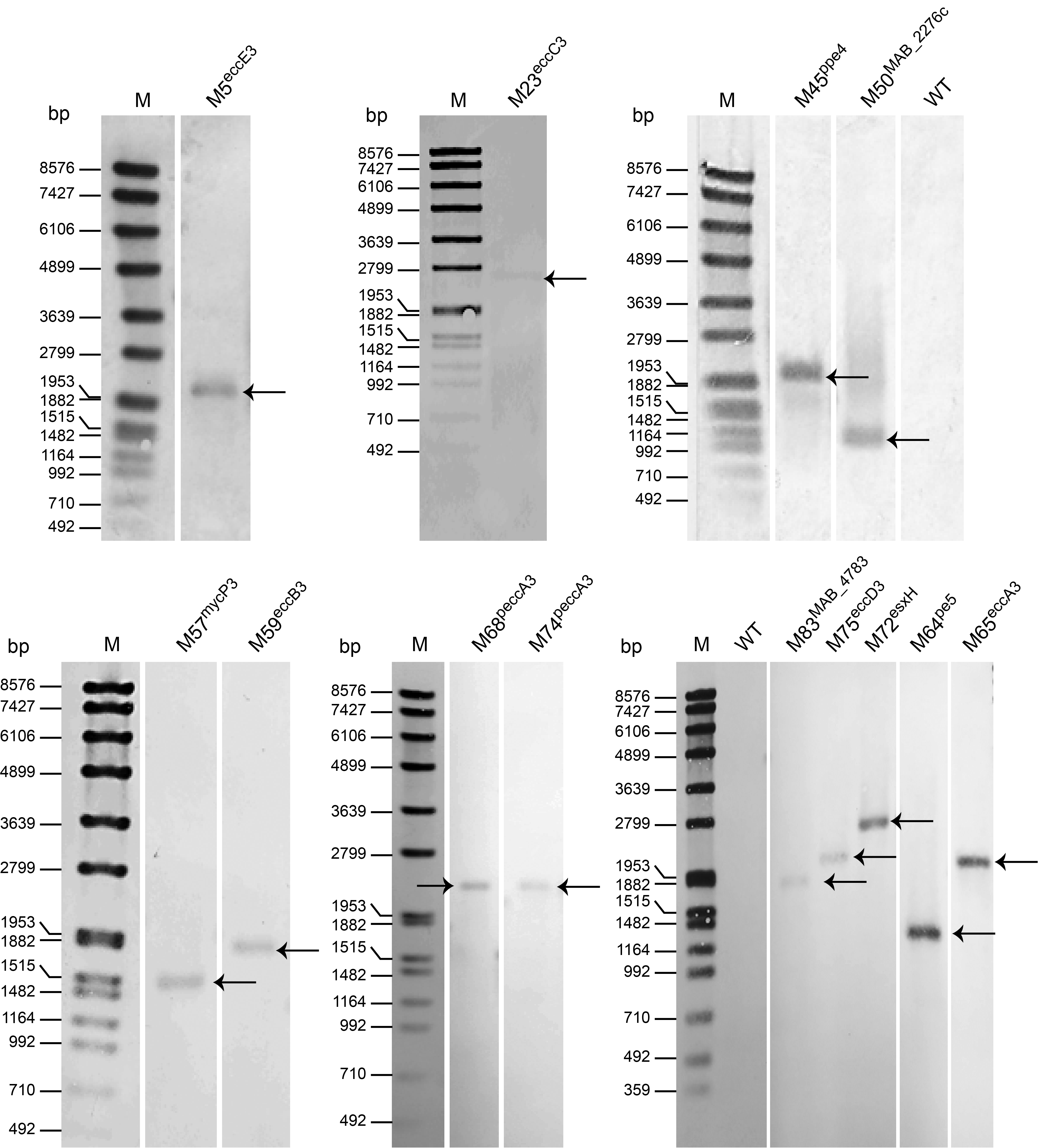

Supplement: Supplementary file 1 [file pathogens-11-00953-s001.zip › Figure S1.tif]

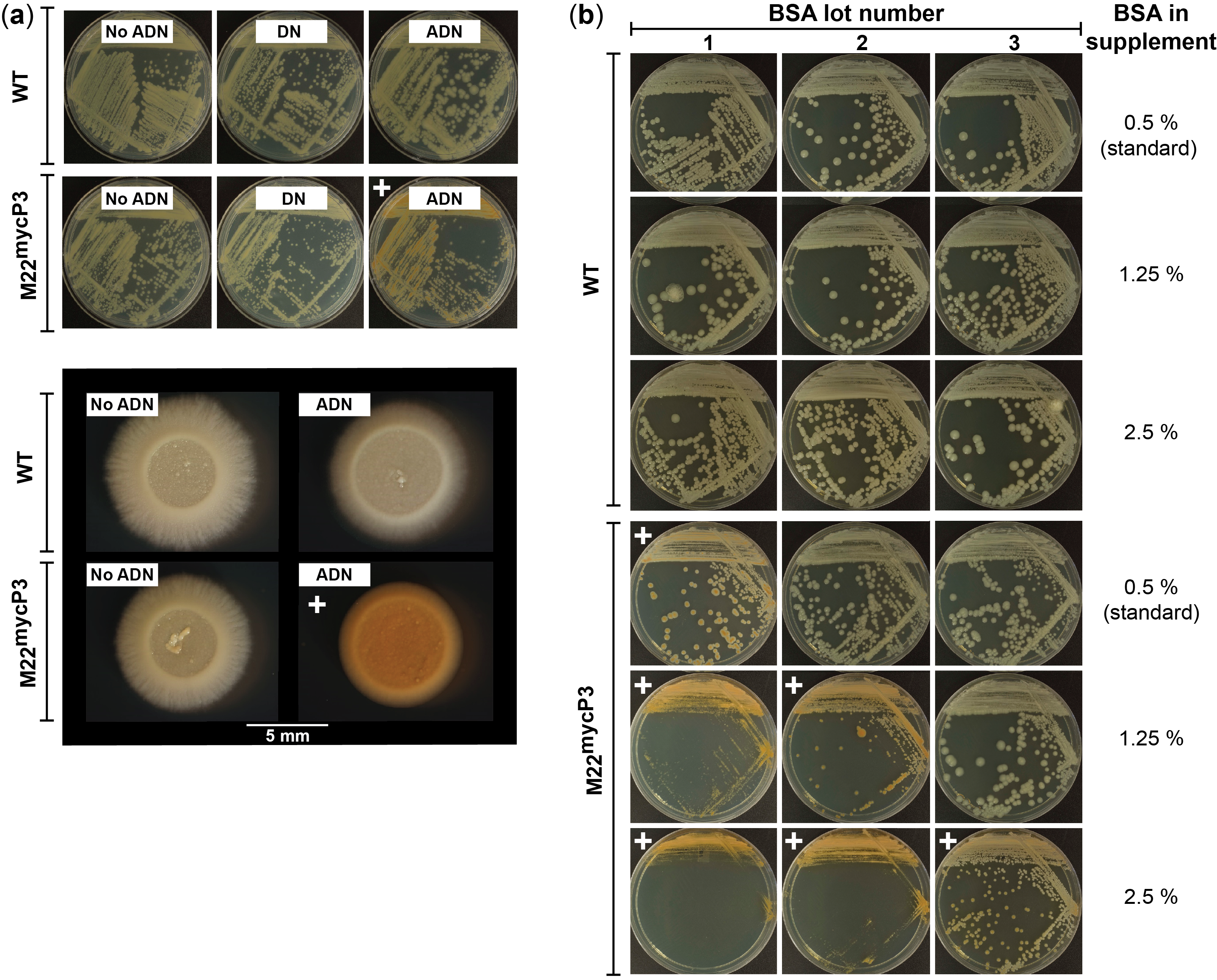

Supplement: Supplementary file 1 [file pathogens-11-00953-s001.zip › Figure S2.tif]

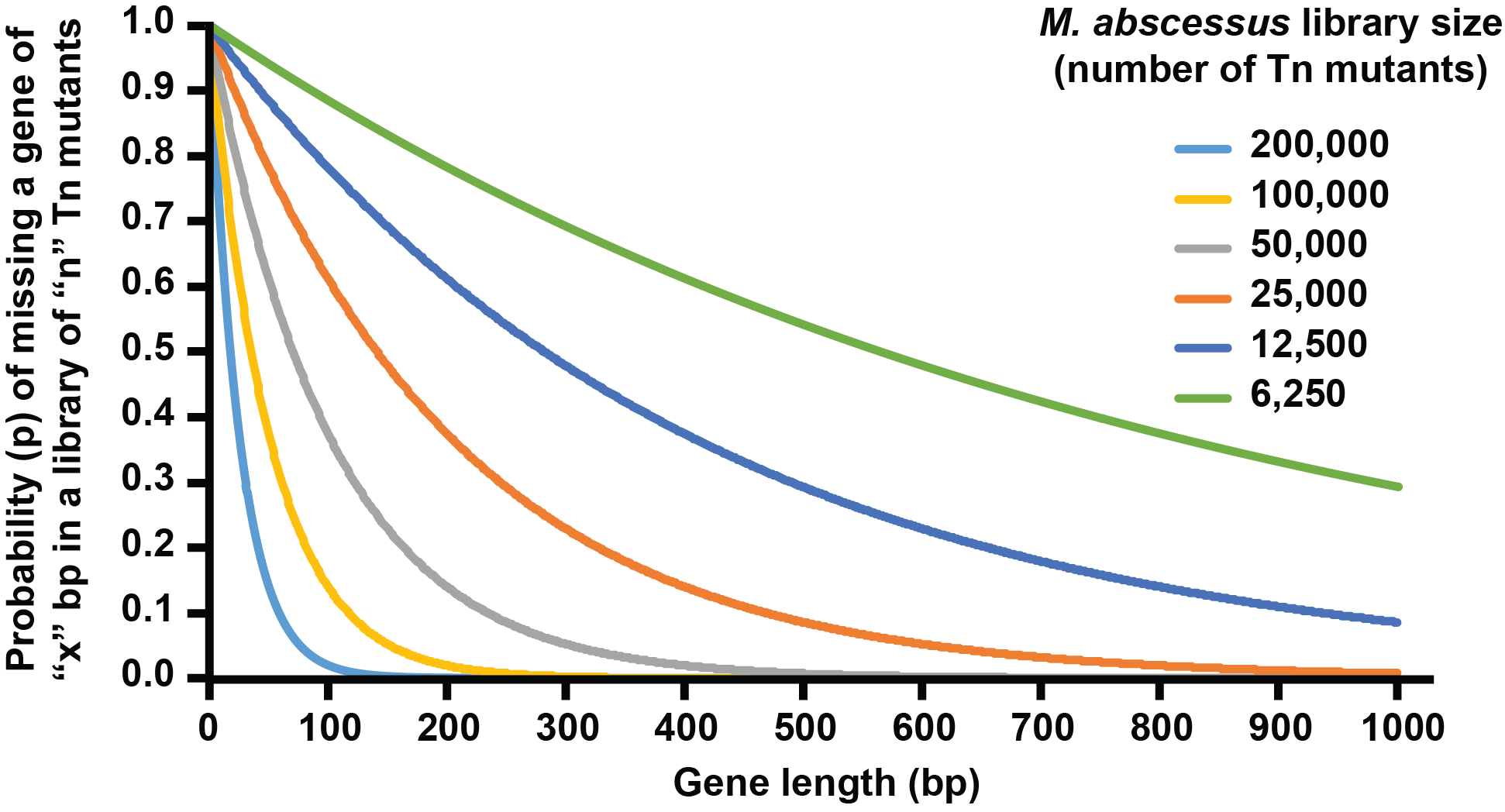

Supplement: Supplementary file 1 [file pathogens-11-00953-s001.zip › Figure S3.tif]

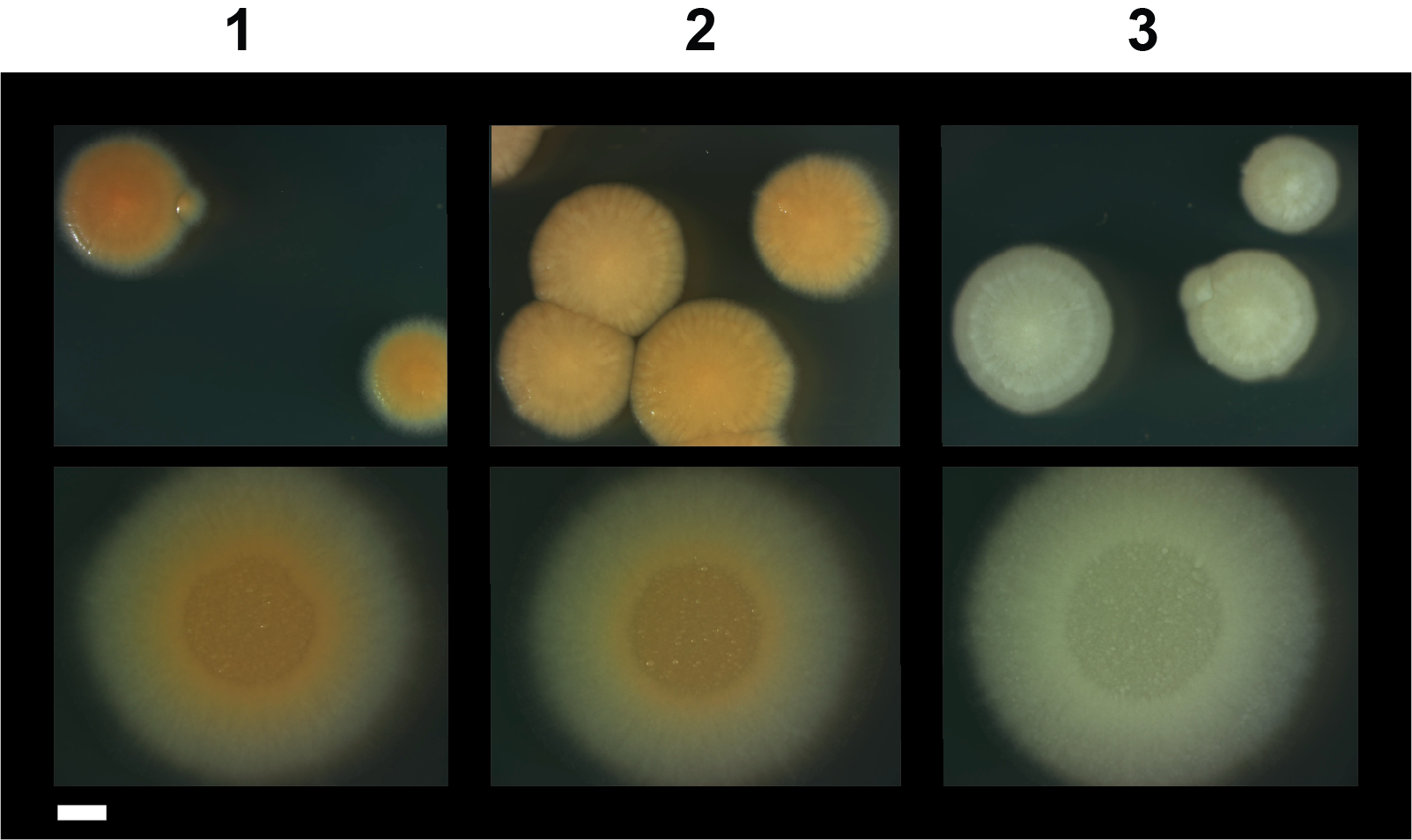

Supplement: Supplementary file 1 [file pathogens-11-00953-s001.zip › Figure S4.tif]

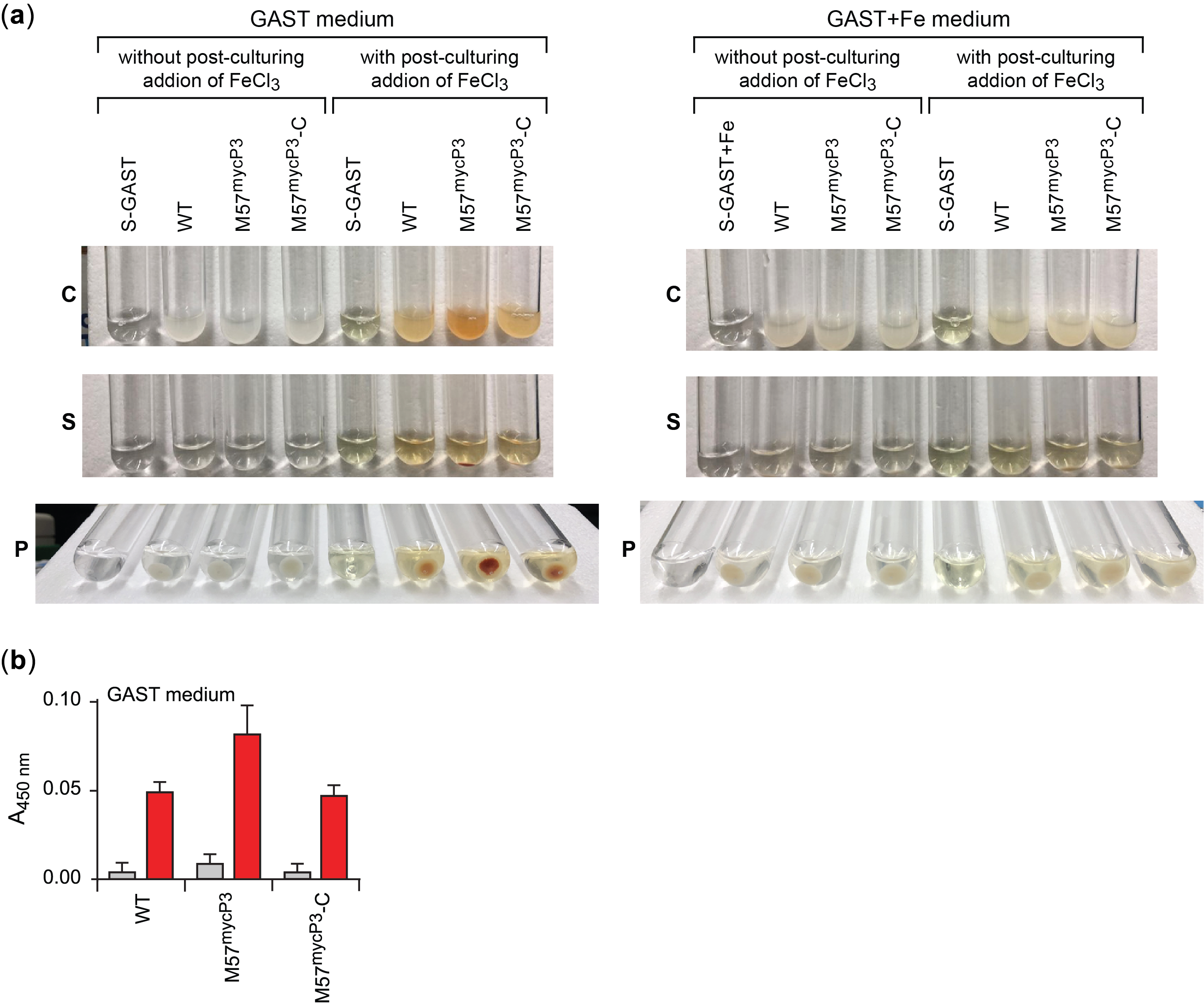

Supplement: Supplementary file 1 [file pathogens-11-00953-s001.zip › Figure S5.tif]

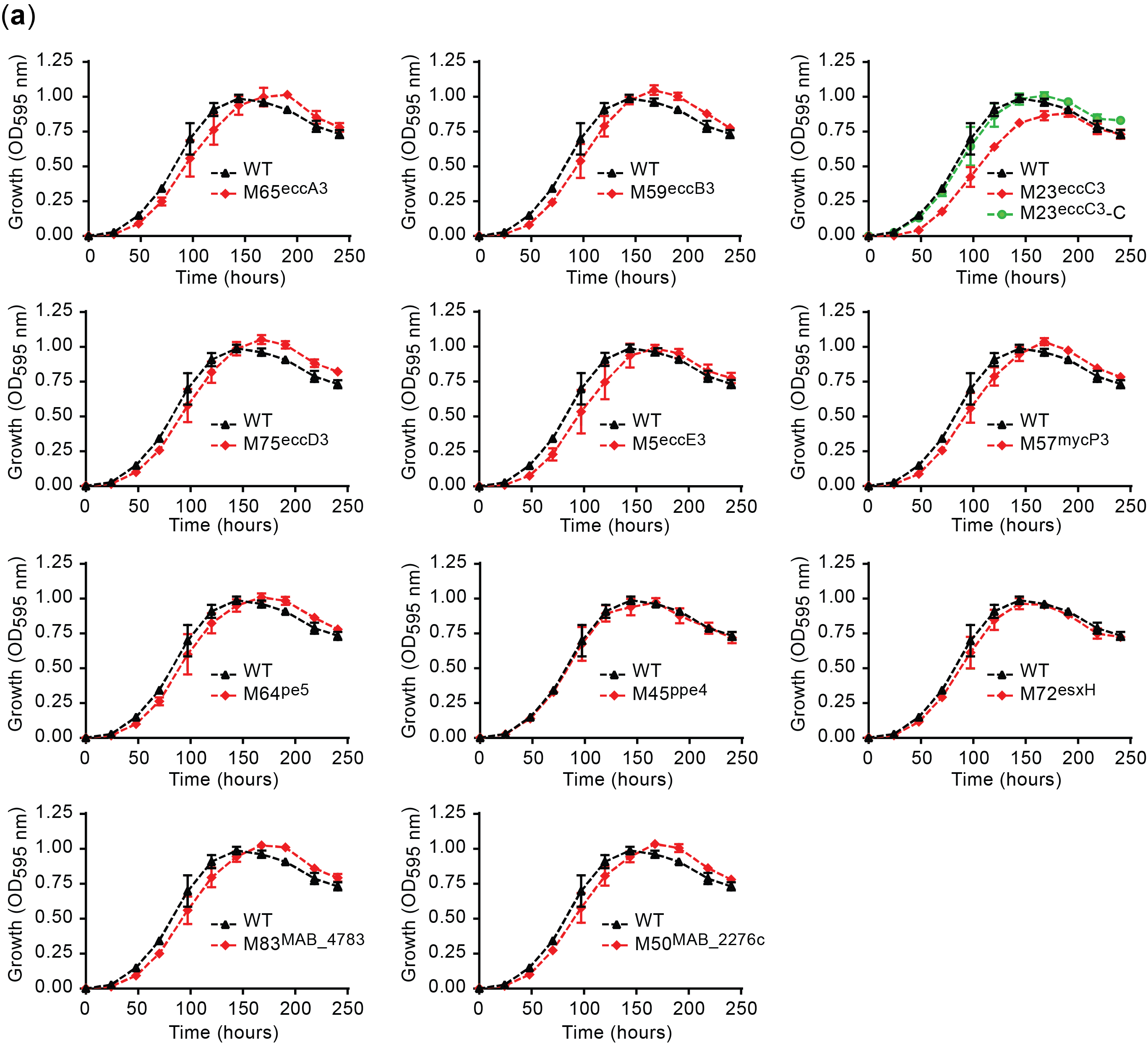

Supplement: Supplementary file 1 [file pathogens-11-00953-s001.zip › Figure S6a.tif]

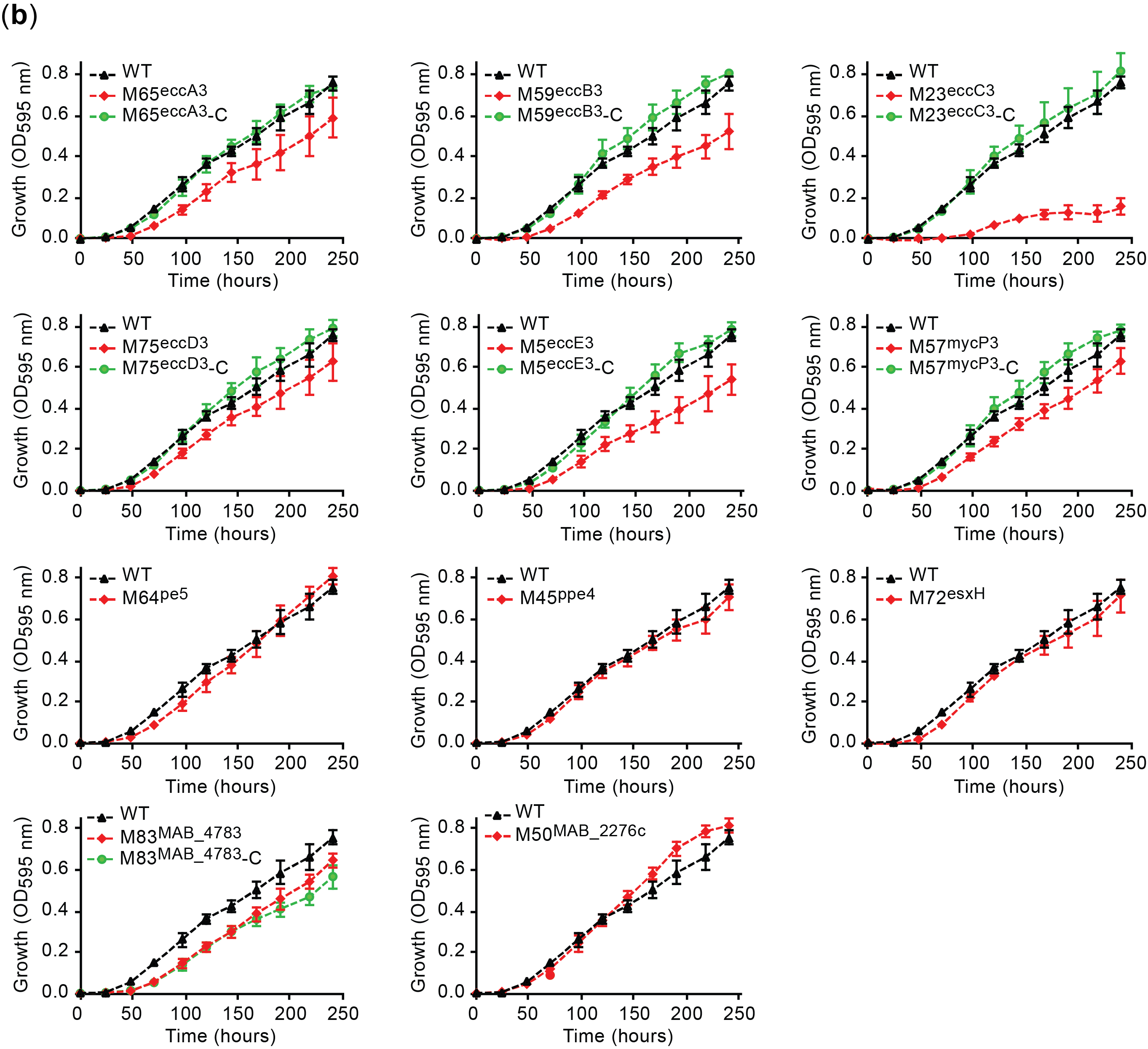

Supplement: Supplementary file 1 [file pathogens-11-00953-s001.zip › Figure S6b.tif]

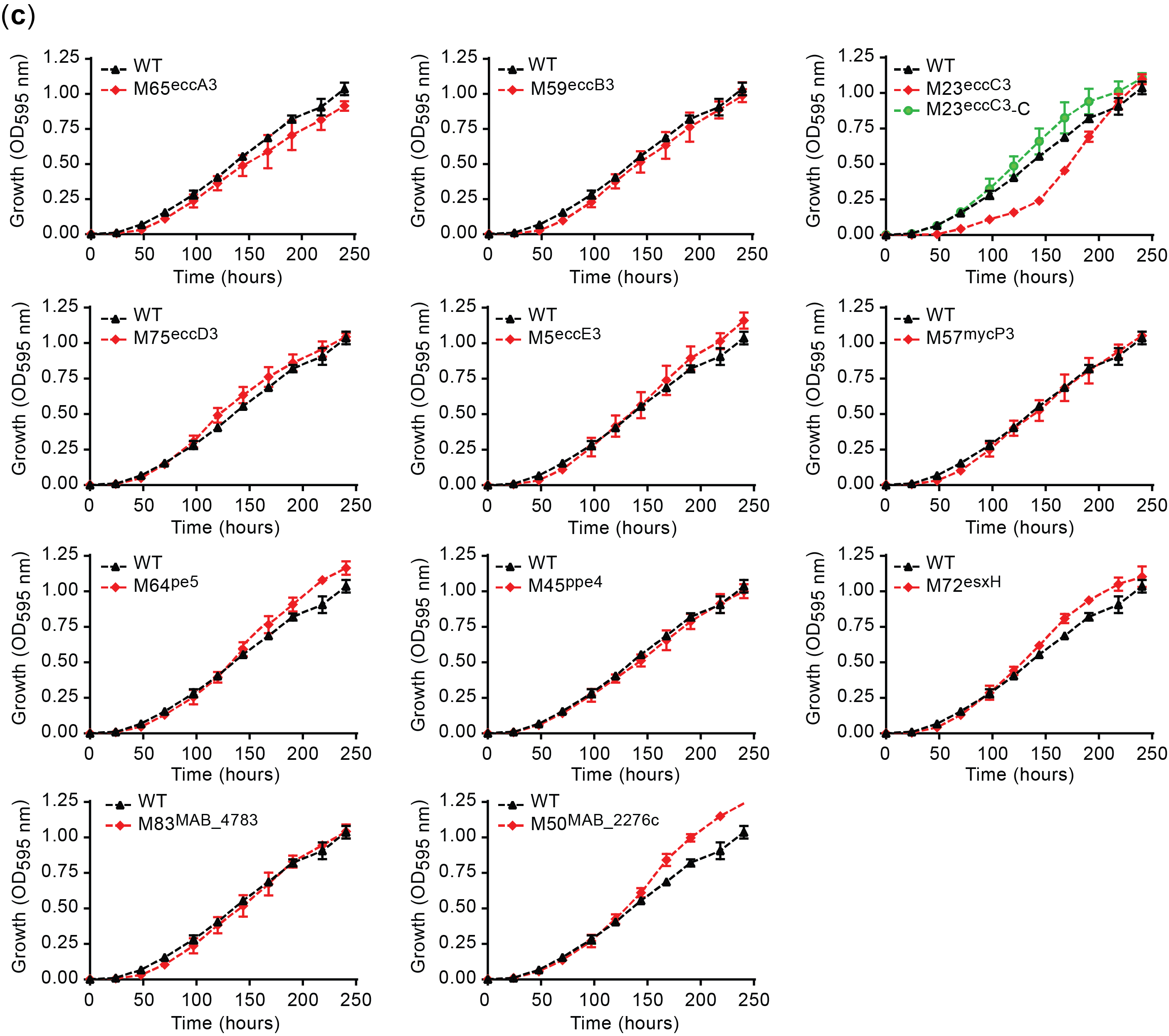

Supplement: Supplementary file 1 [file pathogens-11-00953-s001.zip › Figure S6c.tif]

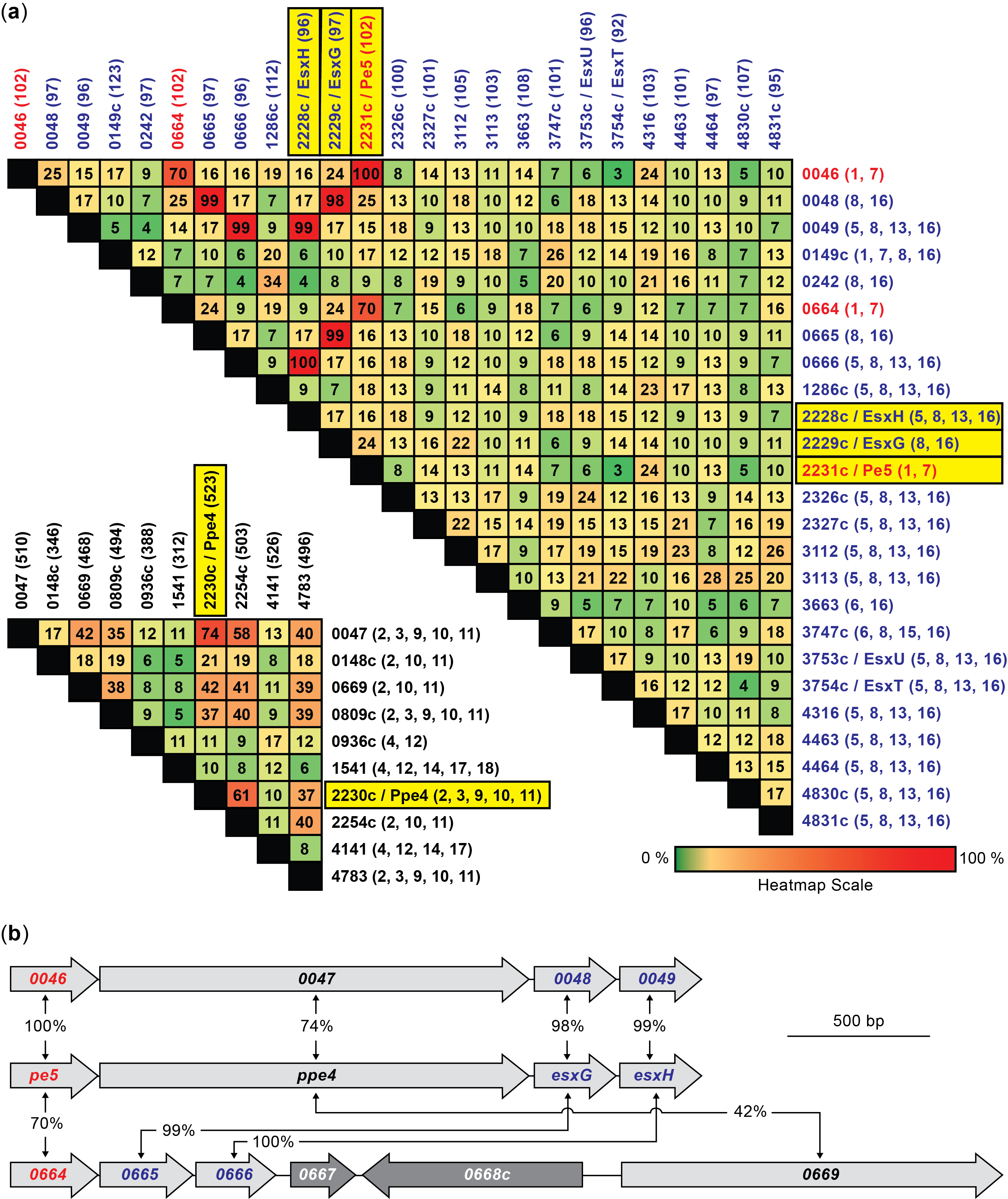

Supplement: Supplementary file 1 [file pathogens-11-00953-s001.zip › Figure S7.tif]

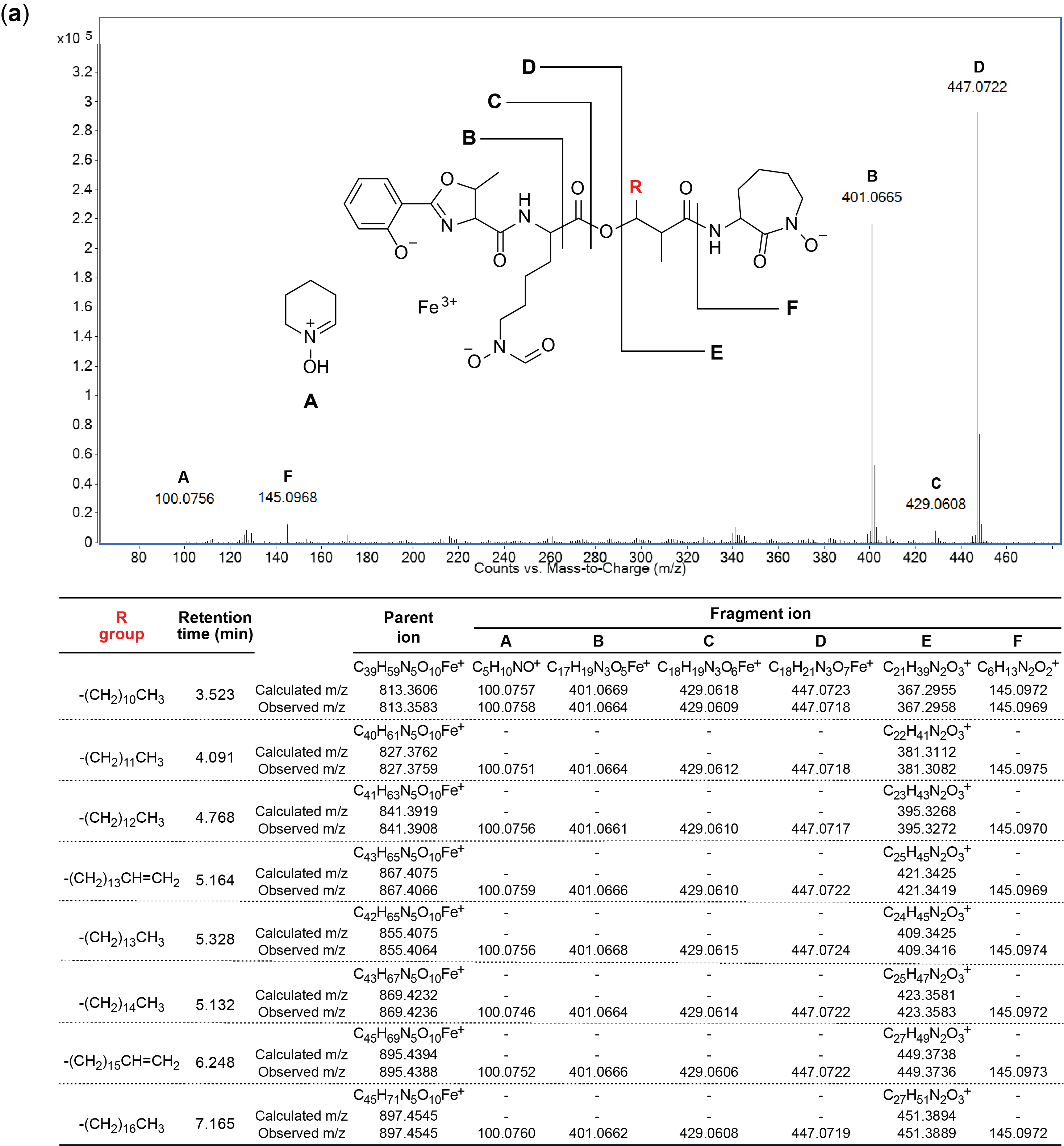

Supplement: Supplementary file 1 [file pathogens-11-00953-s001.zip › Figure S8a.tif]

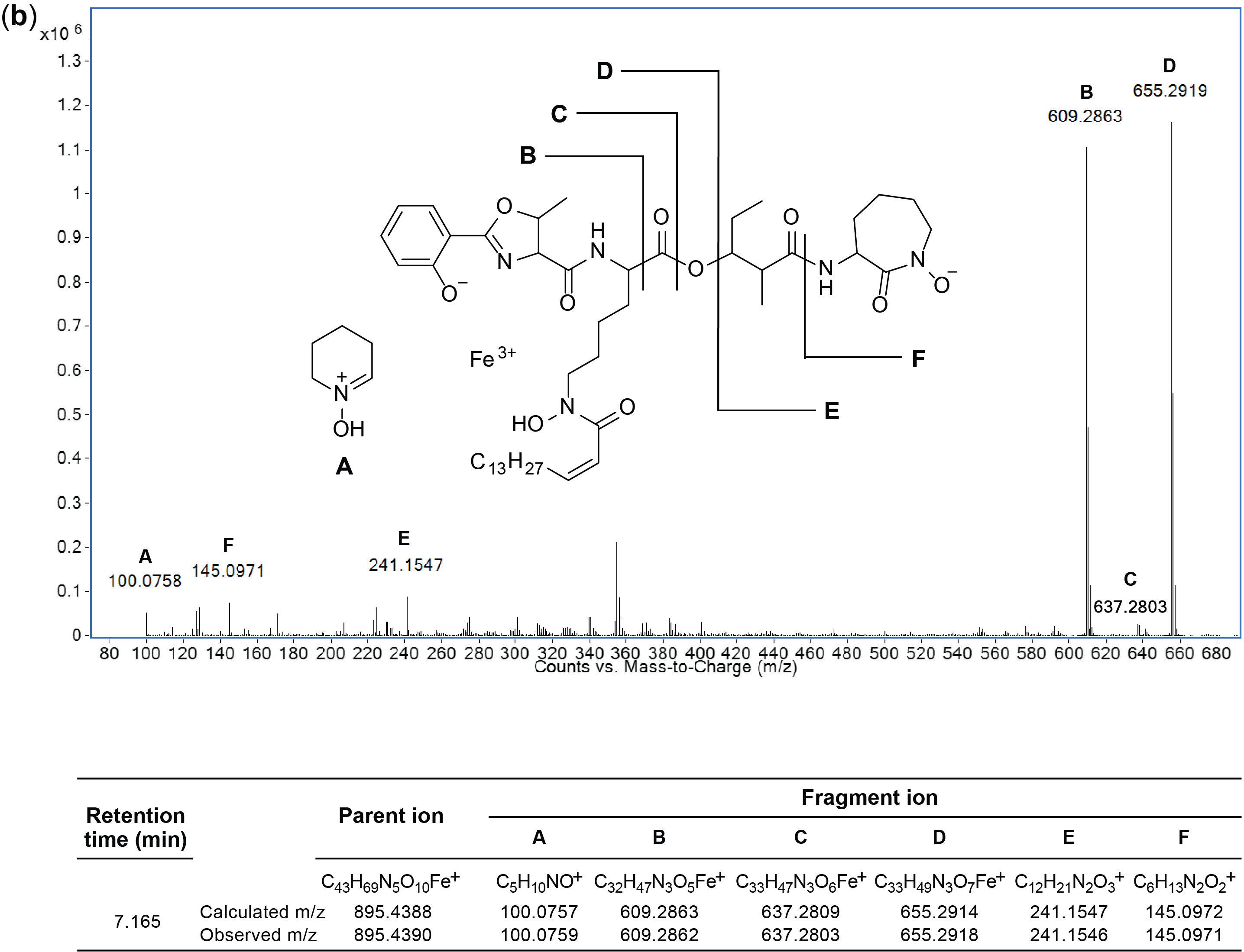

Supplement: Supplementary file 1 [file pathogens-11-00953-s001.zip › Figure S8b.tif]
